# Time-Course Association Mapping of the Grain-Filling Rate in Rice (*Oryza sativa* L.)

**DOI:** 10.1371/journal.pone.0119959

**Published:** 2015-03-19

**Authors:** Erbao Liu, Xiaoli Liu, Siyuan Zeng, Kaiming Zhao, Changfeng Zhu, Yang Liu, Manamik Caleb Breria, Baojuan Zhang, Delin Hong

**Affiliations:** State Key Laboratory of Crop Genetics and Germplasm Enhancement, Nanjing Agricultural University, Nanjing, China; National Institute of Plant Genome Research (NIPGR), INDIA

## Abstract

Detecting quantity trait locus (QTLs) and elite alleles that are associated with grain-filling rate (GFR) in rice is essential for promoting the utilization of hybrid japonica rice and improving rice yield. Ninety-five varieties including 58 landraces and 37 elite varieties from the core germplasm collection were genotyped with 263 simple sequence repeat (SSR) markers. The GFR of the 95 varieties was evaluated at five stages, 7, 14, 21, 28 and 35 days after flowering (DAF) both in 2011 and 2012. We found abundant phenotypic and genetic diversity in the studied population. A population structure analysis identified seven subpopulations. A linkage disequilibrium (LD) analysis indicated that the levels of LD ranged from 60.3 cM to 84.8 cM and artificial selection had enhanced the LD. A time-course association analysis detected 31 marker-GFR associations involving 24 SSR markers located on chromosomes 1, 2, 3, 4, 5, 6, 8, 9, 11 and 12 of rice at five stages. The elite alleles for high GFR at each stage were detected. Fifteen excellent parental combinations were predicted, and the best parental combination ‘Nannongjing62401×Laolaihong’ could theoretically increase 4.086 mg grain^-1^ d^-1^ at the five stages. Our results demonstrate that the time-course association mapping for GFR in rice could detect elite alleles at different filling stages and that these elite alleles could be used to improve the GFR via pyramiding breeding.

## Introduction

Rice (*Oryza sativa* L.) is a globally important cereal crop and is grown on 132 million hectares annually [1]. Although rice yield has increased in recent decades mainly due to genetic improvement [2], higher productivity is needed to meet the rapid population increase, especially with the reduction of arable land and water [3, 4]. The rice yield trait consists of several key components, including grain weight, grain size, grain number, panicle number, and days to heading [5]. And a few QTLs and genes of rice yield related, such as *GS3*, *GS5*, *GW2*, *GW5*, *GW8*, *GL3*, *Gdh7*, and *DTH7* were isolated recently [6–17]. Among all rice yield related traits, grain-filling is a complicated and dynamic process determining the final grain yield [18]. In China, hybrid rice has made great contributions to increasing yield since 1976. Compared to conventional rice, the yield of hybrid rice can increase up to 20% [19]. Although the acreage of hybrid indica rice accounts for approximately 80% of the total area of indica rice, hybrid japonica rice only accounts for approximately 5% of the total area of japonica rice in China. One major reason is that the grain-filling and grain plumpness in hybrid japonica rice are poor on large panicles, which is the main manifestation of heterosis of F_1_ hybrid rice [20–22]. Poor grain-filling and inferior grain plumpness result in decreases of not only the grain yield but also the commodity value [23, 24]. Therefore, improving grain-filling will provide new opportunity to increase grain productivity of F_1_ hybrid rice.

Despite its importance, only several studies have addressed rice grain-filling in the last decade [25–27]. Among the grain-filling related QTLs and genes, the *grain incomplete filling 1* (*GIF1*) is a key regulator of sucrose transport and unloading [25], which encodes a cell-wall invertase required for carbon partitioning during early grain-filling. Using the time-related mapping method, Toshiyuki et al. [26] detected two major QTLs on chromosomes 8 and 12 that were strongly associated with increased filling percentage per panicle. They also reported QTLs of days to heading, accumulated non-structural carbohydrate (NSC) and leaf nitrogen content. In another study, Jia et al. [27] mapped ten additive QTLs for the grain-filling rate using a recombination inbred line (RIL) population that was derived from a cross between Milyang 46 (small grain) and FJCD (large grain) (F_10_ generation). Other studies about sucrose synthase (SUS), starch synthase (SS) and ADP-Glc pyrophosphorylase (AGP) [28–31] revealed that the genes (such as *SUS1*, *SUS2*, *SUS3*, *GBSS*, *OsAPL2* etc.) were the key factors regulating the starch synthase during grain-filling process.

Association mapping using diverse germplasm resources in rice is a new and powerful tool for the elite allele dissection of complex quantitative traits [32–35]. Agrama et al. [32] detected 25 marker-trait associations using yield data and the components of 92 rice germplasm accessions and 123 SSR markers, suggesting that association mapping in rice is a viable alternative to QTL mapping based on crosses between different lines. To our knowledge, there is not report on the GFR of japonica rice using time-course association mapping. Here, we report marker loci that are significantly associated with GFR at five stages (7, 14, 21, 28 and 35 DAF) using time-course association mapping with 263 SSR markers and a core collection of 95 japonica rice accessions.

## Materials and Methods

### Plant materials

Of the 95 diverse rice accessions, 58 were landraces (1–58) from a core germplasm collection that was constructed by Jin et al. [36], and the remaining 37 (59–95) were newly released cultivars. The 95 accessions were collected from six provinces in China (S1 Table).

### Field experiment and measurement

The experiment was conducted at Jiangpu Experimental Station, Nanjing Agricultural University, Nanjing, China, in 2011 and 2012. The seeds of 95 rice accessions were sown in the seedling nursery on 15 May, and the seedlings were transplanted with one seedling per hill on 15 June with three replications. Each plot consisted of five rows with eight hills per row, and the hill spacing was 17 cm×20 cm.

Twenty flowers bloomed on the same day, and five plants from each plot were marked with a black color magic pen (product code, 00633385, ML-T1, made in Japan, http://www.guitar-mg.co.jp/Japan) on the glume surface. Seven days after marking, the marked fresh grains of one plant in each plot were picked and dried in an oven at 105°C to a constant weight. Then, the dried grains were hulled by hand, and five randomly selected grains of brown rice were weighed on a balance with precision up to 0.001 gram and averaged across three replications. Similarly, the grains at 14, 21, 28 and 35 days after flowering were harvested, dried, hulled and weighed. The GFR at each stage was calculated as follows:

$${GFR}_{i}=\frac{{GW}_{i}-{GW}_{i-1}}{7}(i=1,2,3,4,5)$$

where *GFR*_*i*_ is the grain-filling rate (mg grain^-1^ d^-1^), and *GWi* is the grain weight (mg grain^-1^) at stage i.

### SSR marker genotyping

Genomic DNA was extracted from leaf tissue following the methods that are described in Cheng et al. [37]. A total of 263 SSR markers that were selected from the rice maps [38, 39] were used to genotype 95 rice accessions. PCRs were conducted in a 10-μL reaction mixture containing 1 μL of 20 ng μL^-1^ template DNA, 0.6 μL of 25 mmol L^-1^ MgCl_2_, 0.7 μL of 2 pmol μL^-1^ forward primers, 0.7 μL of 2 pmol μL^-1^ reverse primers, 0.2 μL of 2.5 mmol L^-1^ dNTP, 1 μL of 10×PCR buffer, 0.1 μL of 5 U μL^-1^ Taq DNA polymerase (Dongsheng Biotech, China) and 5.7 μL of ddH_2_O. DNA amplification was performed using a PTC-100 Peltier Thermal Cycler (MJ Research Inc., USA). The PCR reaction program included denaturation at 95°C for five minutes, followed by 31 cycles of 95°C for 30 s, 55°C for 30 s, and 72°C for 30 s, and a final extension step at 72°C for five minutes. Electrophoresis and silver staining were performed as described in Liu et al. [40].

### Phenotypic data analysis

The phenotypic data were statistically analyzed using Microsoft Excel 2010. The genotypic and environmental variances of the traits were estimated using the general linear model (GLM) procedure based on multiple environments. The variances were then used to estimate the broad-sense heritability [41] using the following formula: *H* ^2^_B_ = σ^2^_g_/ (σ^2^_g_+σ^2^_e_/n), where σ^2^_g_ is the genetic variance, σ^2^_e_ is the error variance, and n is the number of replicates.

### Genotypic data analysis

The number of alleles per locus, gene diversity and polymorphism information content (PIC) were analyzed using PowerMarker V3.25 software [42]. The LD coefficient (D') of all markers in pairs was evaluated using the software TASSEL 2.1 [43].

$$\;D'=\sum_{i=1}^{u}\sum_{j=1}^{v}p_{i}q_{j}\left|{D'}_{ij}\right|\;{D'}_{ij}=\frac{D_{ij}}{D_{\max}}\;D_{ij}=x_{ij}-p_{i}q_{j}\;D_{\max}=\left[{}_{\min\left[p_{i}\left(1-q_{j}\right),\left(1-p_{i}\right)q_{j}\right],D_{ij}>0}^{\min\left[p_{i}q_{j}\left(1-p_{i}\right)\left(1-q_{j}\right)\right],D_{ij}<0}\right]$$

where *u* and *v* represent the allelic variation of two loci; *p*_*i*_ and *q*_*j*_ represent the frequency of the i allelic variation of locus A and the frequency of the j allelic variation of locus B, respectively; *x*_*ij*_ represents the frequency of gamete *A*_*i*_ *B*_*j*_; and *p*_*i*_ and *q*_*j*_ represent the frequency of allelic variation *A*_*i*_ and *B*_*j*_, respectively. The theory D' value ranges from 0 to 1. A D' value less than 0.5 indicates LD decay.

The decay of LD (with distance in cM) between the SSR loci within the same chromosome was evaluated. The model-based program STRUCTURE 2.2 [44] was used to determine the population structure. Twenty independent runs were performed for each k (from 2 to 10) using a burn-in length of 50,000, a run length of 100,000 and a model for the admixture and independent allele frequency. The true number of populations (K) was often identified using the maximal value of L (K) that was returned by STRUCTURE. A neighbor-joining tree was built by PowerMarker 3.25 based on Nei's genetic distance [45] as calculated by the allele frequencies. The tree was constructed using the MEGA 5.0 software. The coefficient of genetic differentiation (*F*_ST_) was calculated to measure the fixation of different alleles in different populations using the method that was proposed by Weir and Hill [46], and the computing process was completed using the software Arlequin 3.11 [47].

### Association analysis

The general linear model (GLM) in the TASSEL 2.1 software was used for association mapping. The population structure (*Q*) was included as a covariate in the model to test for marker-trait association [48]. The false discovery rate (FDR) was calculated according to method that was proposed by Benjamini et al. [49] to control the expected proportion of falsely rejected hypotheses, and it is the desirable control against errors originating from multiplicity. The allelic effects were estimated compared to the ‘null allele’ (non-amplified alleles) for each locus [50]. The formula that was used to calculate the average positive (negative) allelic effects (AAE) within a locus was

AAE=∑acnc

where *a*_*c*_ represents the phenotypic value of the c^th^ allele with a positive (negative) effect, and *n*_*c*_ represents the number of alleles with positive (negative) effects within the locus.

## Results

### Temperatures during rice grain-filling seasons

**Fig 1:**
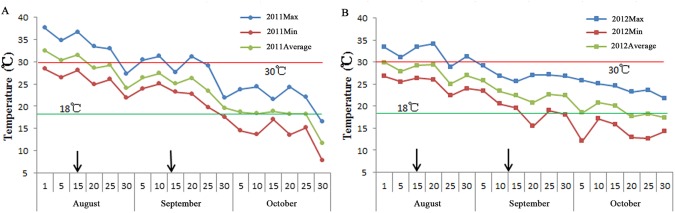
Air temperatures during the grain-filling periods at Jiangpu Experimental Station. (A) In 2011. (B) In 2012. Each datum represents the mean of five days. Two arrows show the earliest heading date and the latest heading date among the 95 rice accessions. The red line and green line show the highest critical temperature and the lowest critical temperature for rice grain-filling, respectively.

In 2011, the variety Yangguang200 headed on August 12, which was the earliest heading date (HD), and the varieties Huangshanshi and Manyedao headed on September 13, which was the latest HD. The corresponding dates were August 13 and September 10, 2012. The daily maximum, minimum and average air temperatures at Jiangpu Experiment Station throughout the periods of grain-filling of the 95 accessions in 2011 and 2012 are shown in Fig. 1. The daily average temperatures during the rice grain-filling were between 18°C and 30°C, which were under the normal climate conditions for rice grain-filling [51]. The changes in the temperatures during rice grain-filling seasons in both years were similar. These data indicate that the grain-filling among 95 rice accessions proceeded under normal and similar climate conditions in both years.

### Phenotypic variation

**Fig 2:**
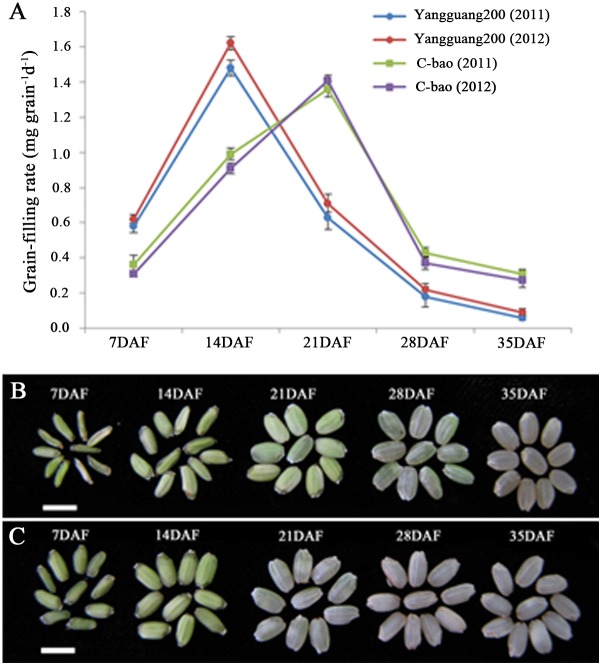
GFR and morphological changes of Yangguang200 and C-bao at five stages. (A) GFR of Yangguang200 and C-bao at five stages in 2011 and 2012. (B) Morphological changes in brown rice in C-bao in 2012. (C) Morphological changes in brown rice in Yangguang200 in 2012. Bar = 5 mm.

There were significant differences in the GFR among the varieties at five stages in both 2011 and 2012, with the CV ranging from 36.49% to 118.26% (Table 1). A high value of broad sense heritability was observed for each stage. The means of GFR over 95 accessions at 14 DAF were 1.01 and 1.11 mg grain^-1^ d^-1^ in 2011 and 2012, respectively, which were the highest among five stages. Among the 95 accessions, the GFR of sixty-two varieties peaked at 14 DAF and that of thirty-three varieties peaked at 21 DAF (S1 Table). Thus, the 95 accessions could be divided into two groups, group 1 corresponding to 62 varieties and group 2 to the 33 aforementioned varieties, according to the date that the GFR peaked. Using variety Yangguang200 (No. 91) to represent group 1 and variety C-bao (No. 94) to represent group 2, the changes in the GFR at 5 stages in both groups are shown in Fig. 2. The GFR of Yangguang200 was higher than that of C-bao at 7 DAF and 14 DAF, and the situation was reversed after 14 DAF. These results indicate that the varieties in group 1 had a faster GFR than that of the varieties in group 2 during the early stages of grain-filling, while the varieties in group 2 had a faster GFR than that of the varieties in group 1 during the late stages of grain-filling. Ninety-five rice varieties also had significant variation in the mature brown rice weight (BRW). Variety Huangsandannuo (No. 58) had the smallest BRW (16.4 mg), and variety H35 (No. 70) had the largest BRW (29.2 mg) (S1 Table).

**Table 1:** Descriptive statistics for the GFR (mg grain^-1^ d^-1^) of 95 rice varieties at 5 stages, mean square among accessions, mean square of error and F value of ANOVA.

Stage	7 DAF		14 DAF		21 DAF		28 DAF		35 DAF	
Year	2011	2012	2011	2012	2011	2012	2011	2012	2011	2012
**Mean**	0.59	0.52	1.01	1.11	0.80	0.83	0.34	0.46	0.19	0.17
**Minimum**	0.29	0.15	0.06	0.02	0.02	0.01	0.00	0.00	0.00	0.00
**Maximum**	1.73	1.16	1.86	2.02	2.48	2.33	1.78	1.71	0.99	1.00
**SD**	0.22	0.23	0.42	0.40	0.42	0.42	0.31	0.40	0.21	0.21
**CV(%)**	37.87	45.13	42.76	36.49	52.80	50.48	90.66	87.95	107.11	118.26
***H*** ^***2***^_**B**_	87.50	96.80	92.64	92.79	93.38	89.51	96.79	87.9	96.61	94.37
***MSv***	0.30	0.23	1.22	0.55	1.71	0.56	1.70	0.78	1.02	0.38
***Mse***	0.02	0.003	0.02	0.01	0.02	0.02	0.01	0.03	0.01	0.01
***F***	17.05**	91.95**	76.53**	39.58**	85.62**	26.59**	181.88**	22.90**	171.94**	51.25**

** indicates significance at the α = 0.01 probability level.

### Genetic diversity

A total of 263 SSR markers were used to measure the genetic diversity of the population. The average number of alleles per SSR locus was 5.93, ranging from 2 to 19. The average genetic diversity throughout all of the SSR loci was 0.5265, ranging from 0.0208 (RM433) to 0.8915 (RM7545). The average PIC value was 0.4776, ranging from 0.0206 (RM433) to 0.8841 (RM7545) (S2 Table).

### Linkage disequilibrium

**Fig 3:**
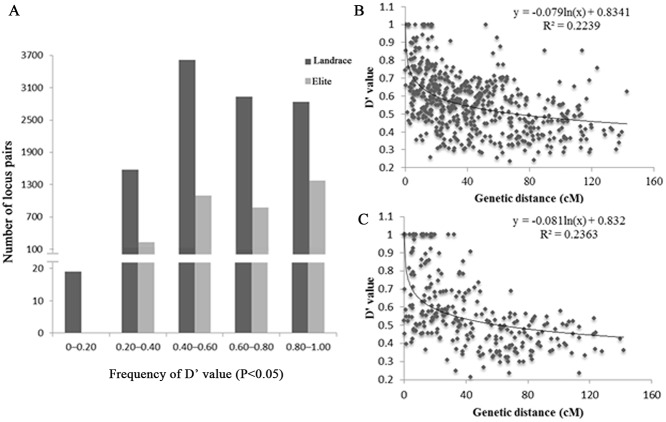
Linkage disequilibrium analysis for landraces and elite varieties. (A) Frequency of D′ value in landrace and elite. (B) Relationship between the D′ value and genetic distance of syntenic marker pairs in population of landraces. (C) Relationship between the D′ value and genetic distance of syntenic marker pairs in population of elite varieties.

A linkage disequilibrium analysis was performed for landraces and elite varieties. The frequency of D' value was showed in Fig. 3A.The average D' value was lower in the landraces (0.640) than in the elite varieties (0.713), suggesting that hybridization and artificial selection increased LD during breeding. The regression analysis of the D' value and the genetic distance of syntenic marker pairs (marker pairs on the same chromosome) demonstrate that the minimum distance of LD decay (D'<0.5) of the landraces and elite varieties is 84.8 cM and 60.3 cM, respectively and that the extent of LD decay is less in landraces than in elite varieties (Fig. 3B and 3C).

### Population structure

**Fig 4:**
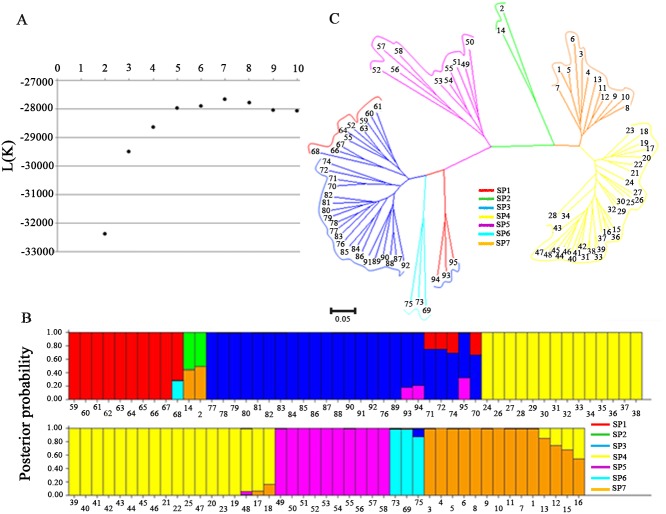
Population structure analysis in 95 rice accessions. (A) Changes in the likelihood value. (B) Posterior probability of 95 accessions belonging to seven subpopulations. (C) Neighbor-joining tree for the 95 accessions using Nei’s genetic distance.

**Fig 5:**
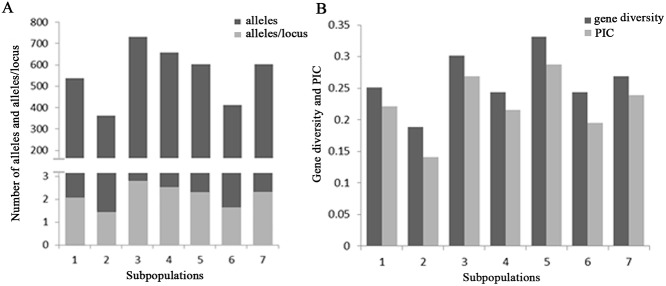
Gene diversity analysis for the seven subpopulations. (A) Number of alleles and alleles/locus in the seven subpopulations. (B) Gene diversity and PIC value in the seven subpopulations.

The Bayesian model-based simulation of the population structure showed that the L (K) value increased with the increase in k and peaked at K = 7 (Fig. 4A). This result indicates that the population that was used in this study is a mixed population consisting of seven subpopulations. The posterior probability value of each variety belonged to seven subpopulations (SP1, SP2, SP3, SP4, SP5, SP6 and SP7) is shown in Fig. 4B. To support the results of the group structure analysis, we constructed a neighbor-joining tree based on Nei's (1983) genetic distance among the 95 rice varieties (Fig. 4C). The neighbor-joining tree showed that the 95 varieties were clearly divided into seven subpopulations. The landraces were divided into four subpopulations, while the elite varieties were divided into three subpopulations. The analysis results based on the STRUCTURE model and the neighbor-joining tree were basically identical. The average *F*_ST_ value among the seven subpopulations was 0.5434 (Table 2). The *F*_ST_ between SP4 and SP7 was the lowest (0.2581), while the *F*_ST_ values between SP1 and SP2 were the largest (0.7071). Among the 7 subpopulations, SP5 had the highest gene diversity of 0.3315, with a total of 603 alleles or 2.30 alleles per locus and a PIC value of 0.2877, followed by SP3 with a gene diversity of 0.3014 and 731 alleles or 2.78 alleles per locus (Fig. 5). However, SP2 had the lowest gene diversity (0.1882) with 1.38 alleles per locus and a PIC value of 0.1412 (Fig. 5).

**Table 2:** Pairwise *F*_ST_ and Nei’s genetic distance among the seven subpopulations.

Subpopulation	1	2	3	4	5	6	7
**1**	—	0.8401	0.3762	0.7167	0.5929	0.5061	0.7165
**2**	0.7071	—	0.8248	0.5106	0.6395	0.8306	0.4674
**3**	0.2766	0.6519	—	0.7334	0.6114	0.5051	0.7252
**4**	0.6817	0.5478	0.6489	—	0.5740	0.7899	0.3209
**5**	0.5057	0.4950	0.4846	0.5336	—	0.6441	0.5770
**6**	0.5078	0.6581	0.4139	0.6972	0.4841	—	0.7884
**7**	0.6517	0.4460	0.6224	0.2581	0.4820	0.6575	—

Nei’s genetic distance is above the diagonal, and the pairwise *F*_ST_ is below the diagonal. All of the *F*_ST_ values are significant (p<0.05).

### Time-course association mapping of GFR

The GLM analysis of the marker-trait association revealed twelve SSR markers that were associated with the GFR (FDR<0.05) at 7 DAF located on chromosomes 2, 3, 4, 5, 6, 8, 9 and 11. The PVE ranged from 8.70% to 22.59%. RM1240_Chr11, residing on 1.46Mb, explained the maximum number of phenotypic variations, viz. 18.59% in 2011 and 22.59% in 2012 (Table 3). RM480_Chr5 had the largest positive AAE, and its effect was 3.6 times as large as the smallest that was observed at 7 DAF (Table 3). Eight markers were associated with the GFR at 14 DAF and were distributed on seven chromosomes; of these markers, RM528_Chr6 had the highest PVE of 25.87% in 2011 and 27.19% in 2012 and the highest positive AAE of 0.505 averaged over both years (Table 3). Two markers were associated with the GFR at 21 DAF and were distributed on chromosomes 5 and 11 (Table 3). The PVE of RM224_Chr11 was slightly larger than that of RM5818_Chr5. Five markers that were associated with the GFR at 28 DAF were detected on chromosomes 2, 4, 5 and 8. RM525_Chr2 had the highest positive AAE of 0.412 at 21 DAF (Table 3). Four markers were associated with the GFR at 35 DAF and were distributed on chromosomes 2, 3, 9 and 12; of these markers, RM1013_Chr9 had the highest PVE and positive AAE (Table 3). Of the 24 SSR markers that were associated with the GFR in both years, seven SSR markers (RM480, RM5818, RM525, RM6361, RM6314, RM224 and RM72) were synchronously associated with the GFR at two stages, and the remaining markers were only associated with one stage (Table 3). RM480, RM525, RM6314 and RM72 were associated with the GFR at both 7 DAF and 28 DAF, RM5818 and RM224 were associated with the GFR at 14 DAF and 21 DAF, and RM6361 was associated with the GFR at 7 DAF and 35 DAF.

**Table 3:** Marker-trait associations with *P*-value less than 0.05, their equivalent false discovery rate probabilities (FDR) less than 0.05, the proportion of phenotypic variance explained (PVE) and the average positive (negative) allele effects (AAE) in 2011 and 2012.

Stage	Marker	Start position (bp)	2011			2012			AAE	
			PVE (%)	*P*-value	FDR	PVE (%)	*P*-value	FDR	Positive	Negative
7 DAF	**RM6361**	(2)17136965	17.67	1.6×10^-2^	0.0305	18.99	1.1×10^-2^	0.0234	0.101	-0.078
7 DAF	**RM525**	(2)28292005	11.89	4.8×10^-3^	0.0171	9.05	3.1×10^-2^	0.0391	0.156	-0.078
7 DAF	RM232	(3)6511645	12.71	8.2×10^-3^	0.0207	20.71	2.8×10^-4^	0.0063	0.247	-0.265
7 DAF	RM6266	(3)23624397	12.66	3.1×10^-3^	0.0159	13.68	3.4×10^-3^	0.0109	0.317	-0.172
7 DAF	**RM6314**	(4)18627879	8.70	2.5×10^-2^	0.0402	9.11	2.8×10^-2^	0.0375	0.138	-0.096
7 DAF	RM3170	(5)27796435	13.96	9.7×10^-3^	0.0256	12.44	3.3×10^-2^	0.0422	0.259	-0.106
7 DAF	**RM480**	(5)27155688	12.69	2.8×10^-3^	0.0134	12.62	5.0×10^-3^	0.0156	0.366	-0.056
7 DAF	RM276	(6)6241911	22.36	5.1×10^-4^	0.0085	17.85	1.1×10^-2^	0.0219	0.260	-0.085
7 DAF	RM314	(6)5735892	18.64	2.6×10^-4^	0.0049	20.09	2.7×10^-4^	0.0047	0.275	-0.094
7 DAF	**RM72**	(8)13237895	12.83	2.7×10^-3^	0.0122	18.25	2.7×10^-4^	0.0031	0.192	-0.13
7 DAF	RM3600	(9)17054142	12.85	3.1×10^-2^	0.0427	14.26	2.5×10^-4^	0.0016	0.247	-0.173
7 DAF	RM1240	(11)1464758	18.59	1.4×10^-2^	0.0293	22.59	4.9×10^-3^	0.0141	0.192	-0.181
14 DAF	RM5389	(1)35726691	14.22	8.1×10^-3^	0.0125	18.5	1.4×10^-3^	0.0053	0.227	-0.138
14 DAF	RM1313	(2)11262096	11.92	2.5×10^-2^	0.0417	16.09	4.8×10^-3^	0.0149	0.145	-0.164
14 DAF	RM471	(4)19007714	12.34	9.3×10^-3^	0.0167	13.08	9.1×10^-3^	0.0223	0.402	-0.659
14 DAF	RM349	(4)32718532	10.76	2.2×10^-2^	0.0333	11.61	2.0×10^-2^	0.0351	0.250	-0.359
14 DAF	**RM5818**	(5)29529228	10.54	2.3×10^-2^	0.0375	10.01	3.8×10^-2^	0.0447	0.221	-0.132
14 DAF	RM528	(6)26172237	25.87	2.0×10^-2^	0.0292	27.19	3.0×10^-6^	0.0011	0.505	-0.289
14 DAF	**RM224**	(11)26796502	14.78	4.5×10^-3^	0.0498	24.37	9.1×10^-4^	0.0043	0.149	-0.322
14 DAF	RM309	(12)21521910	20.06	1.2×10^-4^	0.0042	14.91	3.6×10^-3^	0.0106	0.310	-0.084
21 DAF	**RM5818**	(5)29529228	16.07	2.0×10^-3^	0.0028	16.28	1.6×10^-2^	0.0119	0.454	-0.225
21 DAF	**RM224**	(11)26796502	16.45	2.7×10^-2^	0.0278	17.11	4.6×10^-2^	0.0476	0.150	-0.023
28 DAF	RM263	(2)25889828	11.10	2.8×10^-2^	0.0368	9.43	4.5×10^-2^	0.0477	0.309	-0.168
28 DAF	**RM525**	(2)28292005	9.32	3.0×10^-2^	0.0395	15.14	1.6×10^-3^	0.0045	0.237	-0.178
28 DAF	**RM6314**	(4)18627879	8.64	3.9×10^-2^	0.0474	12.81	4.9×10^-3^	0.0136	0.129	-0.217
28 DAF	**RM480**	(5)27155688	10.60	1.6×10^-2^	0.0289	19.56	1.3×10^-4^	0.0023	0.125	-0.307
28 DAF	**RM72**	(8)13237895	9.67	2.6×10^-2^	0.0316	8.46	4.2×10^-2^	0.0432	0.178	-0.195
35 DAF	**RM6361**	(2)17136965	7.48	2.2×10^-2^	0.0300	8.54	1.9×10^-2^	0.0402	0.056	-0.111
35 DAF	RM5475	(3)30376088	16.70	2.6×10^-2^	0.0375	18.68	2.4×10^-2^	0.0413	0.046	-0.117
35 DAF	RM1013	(9)22509929	19.38	2.2×10^-2^	0.0325	18.74	3.9×10^-4^	0.0141	0.070	-0.072
35 DAF	RM511	(12)17442508	12.69	1.0×10^-2^	0.0175	7.52	3.7×10^-3^	0.0337	0.058	-0.043

Bold markers represent that they were associated with two grain-filling stages. Digit in parentheses of the third column is the chromosome number.

### Best alleles for GFR at the 5 stages

Among the alleles whose phenotypic effects ranked within the top three, RM3170–160 from variety ‘Nannongjing62401’ had the largest phenotypic effect at 7 DAF (Table 4). RM528–135 from variety ‘Tongjing 109’ had the largest phenotypic effect value (0.705) at 14 DAF. Variety ‘Shuijingbaidao’ carried RM5818–150, which had the largest phenotypic effect at 21 DAF. The phenotypic effect of RM72–205 from variety ‘Laolaihong’ was the largest at 28 DAF. Variety ‘Zhen9424’ carried RM511–145, who had the largest phenotypic effect at 35 DAF. These elite alleles for grain-filling were expressed at different stages, and their carrier varieties could be used to predict the excellent parental combinations to improve the overall GFR by pyramiding or substitution breeding. And the rest allelic variation of the loci showing positive allele effects on the top three GFR at five stages in the year of 2011 and 2012 are shown in S3 Table.

**Table 4:** Alleles with positive phenotypic effects on top three GFR across two years and typical varieties carrying the allele.

Stage	Locus-allele	Phenotypic effect value			Typical carrier variety
		2011	2012	Average	
7 DAF	RM3170–160	0.588	0.563	0.576	Nannongjing62401
7 DAF	RM6266–145	0.516	0.612	0.564	Nannongjing62401
7 DAF	RM480–195	0.527	0.453	0.490	Wumangzaodao
14 DAF	RM528–135	0.605	0.805	0.705	Tongjing 109
14 DAF	RM528–245	0.509	0.631	0.570	Wumangzaodao
14 DAF	RM309–160	0.262	0.710	0.486	Baoxintaihuqing
21 DAF	RM5818–150	0.596	0.562	0.579	Shuijingbaidao
21 DAF	RM5818–155	0.594	0.471	0.533	Erlibie, Shengtangqing2
21 DAF	RM224–135	0.202	0.428	0.315	Zaoshirihuangdao
28 DAF	RM72–205	0.609	0.406	0.508	Laolaihong
28 DAF	RM263–175	0.670	0.319	0.495	Laolaihong
28 DAF	RM525–145	0.286	0.356	0.321	Wumangzaodao
35 DAF	RM511–145	0.062	0.098	0.080	Zhen9424
35 DAF	RM1013–160	0.104	0.036	0.070	Kaiqing, Diantun502xuanzao
35 DAF	RM511–135	0.038	0.075	0.057	Taijing 9 xuan

### Excellent parental combinations predicted for GFR improvement

Fifteen excellent parental combinations for GFR improvement were predicted (Table 5) based on the data of Table 4 and S4 Table. As shown in Table 3, twenty-four SSR loci were significantly associated with the GFR at 5 stages. Among the 11 SSR loci that were detected in variety Nannongjing62401 (No. 73), 8 loci (3 at 7 DAF, 3 at 14 DAF, 1 at 21 DAF and 1 at 28 DAF) had positive alleles with top phenotypic effects, while 3 loci (1 at 21 DAF and 2 at 28 DAF) had negative effects. In variety Wumangzaodao (No. 6), 7 loci (2 at 7 DAF, 2 at 14 DAF, 1 at 21 DAF and 2 at 28 DAF) had positive alleles with top phenotypic effects, while 4 loci (1 at 7 DAF, 14 DAF, 21 DAF and 28 DAF) had negative effects. By crossing the two varieties, all of the elite alleles at the 11 loci could be pyramided into a plant (variety), and the grain-filling rate of the new variety could be theoretically increased by 3.867 mg grain^-1^ d^-1^. Similarly, the other 14 excellent parental combinations were predicted (Table 5).

**Table 5:** Excellent parental combinations predicted for GFR improvement.

Best predicted parental combination	Each stage GFR improvement predicted (mg grain^-1^ d^-1^)					Total GFR improvement predicted (mg grain^-1^ d^-1^)
	7 DAF	14 DAF	21 DAF	28 DAF	35 DAF	
Nannongjing62401×Laolaihong	1.382	0.873	0.588	1.156	0.087	4.086
Nannongjing62401×Wumangzaodao	1.630	1.204	0.542	0.464	0.027	3.867
Nannongjing62401×Shuijingbaidao	1.382	0.873	0.678	0.783	0.021	3.737
Baoxintaihuqing×Zaoshirihuangdao	0.811	0.615	0.848	1.156	-0.087	3.413
Wumangzaodao×Zaoshirihuangdao	0.352	1.174	0.848	0.951	-0.012	3.313
Baoxintaihuqing×Shuijingbaidao	0.881	0.615	0.678	1.156	-0.087	3.243
Nannongjing62401×Baoxintaihuqing	1.630	1.122	-0.093	0.516	0.027	3.202
Nannongjing62401×Kaiqing	1.382	0.873	0.588	0.166	0.183	3.192
Nannongjing62401×Erlibie	1.382	0.873	0.542	0.296	0.087	3.180
Nannongjing62401×Zhen9424	1.382	0.873	0.542	0.166	0.206	3.169
Nannongjing62401×Taijing 9 xuan	1.382	0.873	0.542	0.166	0.183	3.146
Baoxintaihuqing×Laolaihong	0.881	1.339	0.588	1.156	0.012	3.129
Tongjing 109×Laolaihong	0.018	1.339	0.588	1.156	0.012	3.113
Tongjing 109×Baoxintaihuqing	0.881	1.588	-0.093	0.653	-0.048	2.981
Wumangzaodao×Tongjing 109	0.550	1.339	0.542	0.448	0.027	2.906

## Discussion

### Effects of the variability in HD and BRW on GFR

The variation in crop ontogeny, one of the most essential aspects of the field experiment, can cause environmental variation in their subsequent developmental stages. In terms of the GFR in rice, the variation in HD and BRW may lead to environmental variation, which may significantly influence GFR. In this study, while variability in days to heading was observed among the 95 rice accessions, the climate conditions, especially the average daily temperature, were normal and favorable for rice grain-filling in both years (Fig. 1). We also found that the BRW and GFR of the 95 varieties were independent. For example, ‘Wanhuangdao’ (No. 3) and ‘Guozinuo’ (No. 4) with nearly the same BRW belonged to group 1 and group 2, respectively, while ‘Hongmangshajing’ (No. 2) and ‘Wanhuangdao’ (No. 3) belonged to group 1 with significant differences in the BRW (S1 Table). These results indicate that using 95 core accessions to study the GFR was feasible, even though there was variability in the HD and BRW.

### Importance of the time-course method in studying the GFR

Grain-filling in rice is a critical and dynamic process, which highlights the need for a time-course method. The significant variation in the rice GFR at five stages demonstrates that the dissection of the grain-filling rate by time course is necessary. Grain-filling has also been studied at different stages [25, 26], e.g., Toshiyuki et al. [26] studied the grain-filling related QTLs at four stages. If the average GFR of the entire filling stage instead of the individual stage was considered, we could not detect elite alleles at different stages. Additionally, the study of grain-filling is needed at different stages because of the complexity of the grain-filling process involving various genetic mechanisms [52] and environmental factors [26].

### Comparison of the genetic diversity of the rice core accessions

Conducting a successful association study requires an appropriate sample size and abundant diversity in phenotype and genotype [53]. Although only 95 rice varieties were included in this study, these varieties were core germplasm accessions that were selected from six provinces in China. These accessions have large variation in phenotype, a coefficient of variation ranging from 36.49% to 118.26%, and an average number of alleles per locus over 263 SSR markers of 5.93, which exceeds most of the reported values in other diversity studies of rice germplasm; the average number of alleles per locus in those studies ranged from 3.88 to 5.89 [34, 53–56]. The wide range of genetic diversity makes this collection one of the best for pyramiding elite alleles that associated with the GFR in rice.

### Implication of the population structure and LD in association mapping

Determining the population structure is essential to avoid false-positive results between the phenotype and genotype in association mapping because of the linkage disequilibrium in natural populations [57]. The genetic structure of rice (*O*. *sativa*) has been previously reported [32, 58]. Agrama et al. [32] detected eight subpopulations among 103 rice accessions with 123 SSR markers. Similarly, our study has detected seven subpopulations with 263 SSR markers by STRUCTURE and a neighbor-joining tree. Fifty-eight landraces were divided into four subpopulations, while the elites were divided into three subpopulations. The results that were obtained by STRUCTURE analysis and neighbor-joining clustering were consistent except for the clustering of a few varieties. Linkage disequilibrium is the basis of association analysis [59]. In this study, an LD analysis was conducted between landraces and elite populations, and the genetic distance of decay was 60 cM, which is longer than that of previous studies [32, 34]. Agrama et al. [32] indicated that LD decays at 20–30 cM using SSR markers, and the LD of the germplasm accessions of Jin et al. [34] did not decay until 25–50 cM. These results indicate that the 95 rice accessions in our study experienced more hybridization and artificial selection than did the accessions of other studies. As a self-pollinated crop, the genetic distance of decay of rice was much longer than that of cross-pollinated crops, such as maize, whose LD diminished over a distance of 2000 bp [60].

### Superiority of time-course association mapping in studying the GFR

**Fig 6:**
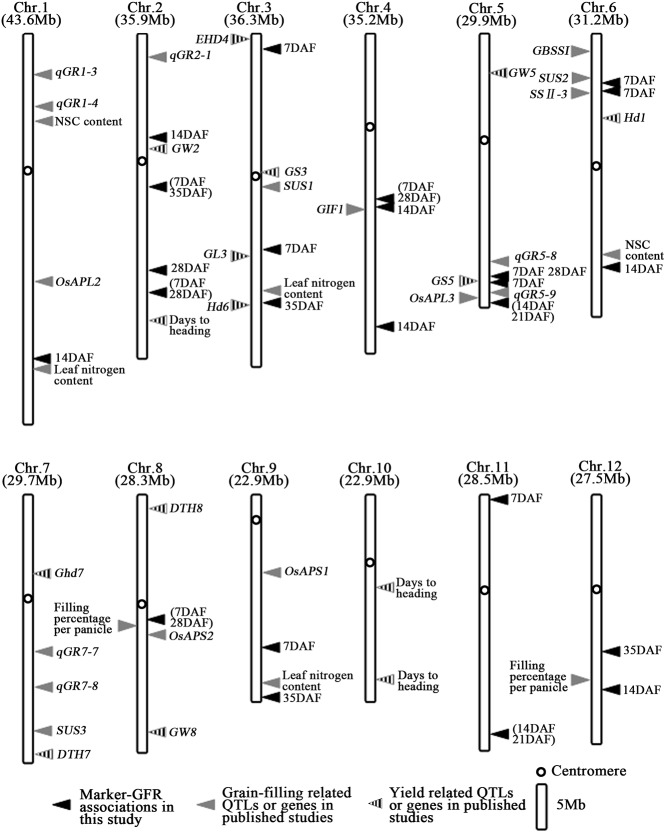
Distribution of marker-GFR and QTLs or genes for grain-filling and yield related on rice chromosome.

Linkage mapping in families and population-based association mapping are the two main approaches to mapping the relevant genes and identifying the variants that are associated with the traits. Linkage mapping mainly identifies only those loci with the strongest influence. However, for complex traits such as GFR, genetic association mapping has greater power than linkage studies to identify variants with weak effects [61]. Using time-course association mapping, we detected 24 SSR markers on chromosomes 1, 2, 3, 4, 5, 6, 8, 9, 11 and 12 that were associated with the GFR both in 2011 and 2012 (FDR<0.05). By comparing with other studies, we found eight of the 24 SSR markers detected in this study were novel, and the other 16 SSR markers were located near to the chromosome regions harboring grain-filling and yield related QTLs or genes which have been reported. Among the sixteen SSR markers, RM6361_Chr2, RM6266_Chr3, RM3170_Chr5 and RM480_Chr5 were near to the chromosome regions containing QTLs or genes for grain size, such as *GW2*, *GL3*, *GS5*, indicating that grain size might have impaction on grain-filling in the grain maturation process. *GL3* was found to have favorable effect not only on grain length but also on grain filling [11]. RM263_Chr3 and RM5475_Chr3 were near to the chromosome regions covering *EHD4* and *Hd6* of heading-related genes shown in Fig. 6, respectively. Marker RM471_Chr4 associated with GFR at 14DAF and RM6314_Chr4 associated with GFR at both 7DAF and 28DAF were near to chromosome region harboring the gene *GIF1* [25] (Fig. 6). RM72_Chr8, RM5389_Chr1, RM5475_Chr3, RM1013_Chr9 and RM528_Chr6 were near to chromosome regions containing the QTLs of filling percentage per panicle, leaf nitrogen content and NSC content detected by Toshiyuki et al [26]. RM314_Chr6, RM276_Chr6 and RM5818_Chr5 were near to chromosome region harboring the starch synthase related genes *SUS 2*,*SSII-3* and *Os APL 3* (Fig. 6). RM480_Chr5, RM3170_Chr5 and RM5818_Chr5 were near to chromosome regions containing the *qGR-5–8* and *qGR-5–9* for GFR [27]. Percentages of phenotypic variations explained by SSR markers detected in our study ranged from 7.48% to 27.19%. These comparisons elucidated that time-course association mapping could not only detect more loci underlying GFR in rice than linkage mapping could, but also detect loci with weak effects.

### Analysis of excellent parental combinations predicted for GFR improvement

Utilization of all the elite alleles that were detected at different filling stages in this study may improve the GFR of F_1_ hybrid japonica rice. Among the fifteen excellent predicted combinations, the best parental combination ‘Nannongjing62401×Laolaihong’ can theoretically improve the GFR by 4.086 mg grain^-1^ d^-1^ at all of the stages (Table 5). Eight of the 15 combinations, especially the top 3, have ‘Nannongjing62401’ as a parent. Therefore, ‘Nannongjing62401’ can be used as an outstanding parent to improve the GFR of F_1_ hybrid japonica rice. Due to the complexity of grain-filling in rice, the performances of all of the excellent predicted parental combinations in rice yield improvement require further verification in rice production.

## Conclusions

Eight novel GFR-associated markers were detected in this study by time-course association mapping with the core rice germplasm with a large variation in phenotype. Pyramiding the elite alleles at different grain-filling stages can significantly improve the GFR of rice. Fifteen excellent parental combinations were predicted, of which ‘Nannongjing62401×Laolaihong’ was the best. Variety ‘Nannongjing62401’ (No. 73) can serve as an elite parent in combination with other varieties, aiming at the genetic improvement of the GFR. The results of this study indicate that time-course association mapping in elite germplasm seems to be a better approach than linkage mapping in mining elite alleles for marker-assisted selection and breeding by the design of GFR in rice.

## Supporting Information

S1 Table Brown rice weight at five filling stages of the 95 rice germplasm accessions, their geographical origin, coordinates and ID code in this study. The number 1 and 2 at the top right of accession name indicate the accession belonged to group1 and group2, respectivly. Bold accession names (No.1–58) are the core germplasm collection constructed by Jin et al., doi: 10.3864/j.issn.0578-1752.2008.11.051. The accessions (No.59–95) can be linked on http://www.ricedata.cn/variety/, http://202.127.42.178:4000/countryseed/SpeciesDemand/Default.aspx and http://icscaas.com.cn/sites/ics/ with the accession ID.
(DOC)

S2 Table Summary statistics for the 263 SSR markers used in this study. (DOC)

S3 Table Allelic variation of the loci showing positive allele effects on the top three GFR at five stages in the year of 2011 and 2012. Bold alleles represent that they were alleles with positive phenotypic effects on top three GFR at each stage across two years.
(DOC)

S4 Table Allelic variation of the loci with average positive allele effects on the top three GFR at five stages. ‘√’indicates the accession with the positive allele; ‘Δ’indicates the accession with the negative allele. The values under alleles are the average positive (negative) allelic effects of the alleles.
(XLS)

## References

[1] Khush GS. What it will take to feed 5.0 billion rice consumers in 2030. Plant Mol Biol. 2005;59: 1–6. 16217597 10.1007/s11103-005-2159-5

[2] Yu YQ, Huang Y, Zhang W. Changes in rice yields in China since 1980 associated with cultivar improvement, climate and crop management. Field Crops Res. 2012;136: 65–75.

[3] Peng SB, Khush GS, Virk P, Tang QY, Zou YB. Progress in ideotype breeding to increase rice yield potential. Field Crops Res. 2008;108: 32–38.

[4] Cassman KG. Ecological intensification of cereal production systems: yield potential, soil quality, and precision agriculture. Proc Natl Acad Sci U S A. 1999;96: 5952–5959. 10339523 10.1073/pnas.96.11.5952 PMC34211

[5] Yano M. Genetic and molecular dissection of naturally occurring variation. Curr.Opin.Plant Biol. 2001;4: 130–135. 11228435 10.1016/s1369-5266(00)00148-5

[6] Fan C, Xing Y, Mao H, Lu T, Han B, Xu C, et al GS3, a major QTL for grain length and weight and minor QTL for grain width and thickness in rice, encodes a putative transmembrane protein. Theor Appl Genet. 2006;112: 1164–1171. 16453132 10.1007/s00122-006-0218-1

[7] Li YB, Fan CC, Xing YZ, Jiang YH, Luo LJ, Sun L, et al Natural variation in GS5 plays an important role in regulating grain size and yield in rice. Nat Genet. 2011;43: 1266–1270. 10.1038/ng.977 22019783

[8] Song XJ, Huang W, Shi M, Zhu MZ, Lin HX. A QTL for rice grain width and weight encodes a previously unknown RING-type E3 ubiquitin ligase. Nat Genet. 2007;39: 623–629. 17417637 10.1038/ng2014

[9] Weng JF, Gu SH, Wan XY, Gao H, Guo T, Su N, at al Isolation and initial characterization of GW5, a major QTL associated with rice grain width and weight. Cell Res. 2008;18: 1199–1209. 10.1038/cr.2008.307 19015668

[10] Wang SK, Wu K, Yuan QB, Liu XY, Liu ZB, Lin XY, et al Control of grain size, shape and quality by OsSPL16 in rice. Nat Genet. 2012;44: 950–955. 10.1038/ng.2327 22729225

[11] Zhang XJ, Wang JF, Huang J, Lan HX, Wang CL, Yin CF, et al Rare allele of OsPPKL1 associated with grain length causes extra-large grain and a significant yield increase in rice. Proc Natl Acad Sci U S A. 2012;109: 21534–21539. 10.1073/pnas.1219776110 23236132 PMC3535600

[12] Xue WY, Xing YZ, Weng XY, Zhao Y, Tang WJ, Wang L, et al Natural variation in Ghd7 is an important regulator of heading date and yield potential in rice. Nat Genet. 2008;40: 761–767. 10.1038/ng.143 18454147

[13] Gao H, Jin MN, Zheng XM, Chen J, Yuan DY, Xin YY, et al Days to heading 7, a major quantitative locus determining photoperiod sensitivity and regional adaptation in rice. Proc Natl Acad Sci U S A. 2014;111: 16337–16342. 10.1073/pnas.1418204111 25378698 PMC4246261

[14] Takahashi Y, Shomura A, Sasaki T, Yano M. Hd6, a rice quantitative trait locus involved in photoperiod sensitivity, encodes the alpha subunit of protein kinase CK2. Proc Natl Acad Sci U S A. 2001;98: 7922–7927. 11416158 10.1073/pnas.111136798 PMC35444

[15] Gao H, Zheng XM, Fei GL, Chen J, Jin MN, Ren YL, et al Ehd4 encodes a novel and oryza-genus-specific regulator of photoperiodic flowering in rice. PLoS Genet. 2013;9: e1003281 10.1371/journal.pgen.1003281 23437005 PMC3578780

[16] Yano M, Katayose Y, Ashikari M, Yamanouchi U, Monna L, Fuse T, et al Hd1, a major photoperiod sensitivity quantitative trait locus in rice, is closely related to the arabidopsis flowering time gene CONSTANS. Plant Cell. 2000;12: 2473–2483. 11148291 10.1105/tpc.12.12.2473 PMC102231

[17] Wei XJ, Xu JF, Guo HN, Jiang L, Chen SH, Yu CY, et al DTH8 suppresses flowering in rice, influencing plant height and yield potential simultaneously1^[W][OA]^ . Plant Physiol. 2010;153: 1747–1758. 10.1104/pp.110.156943 20566706 PMC2923886

[18] Cock JH, Yoshida S. Accumulation of 14C-labelled carbohydrate before flowering and its subsequent redistribution and respiration in the rice plant. Jpn J Crop Sci. 1972;41: 226–234.

[19] Mao CX. Developing indica-type hybrid in China In: Xie F, Hardy B, editors. Accelerating hybrid rice development. Los Banos: International Rice Research Institute; 2010 pp. 581–592.

[20] Virmani SS, Aquino RC, Khush GS. Heterosis breeding in rice (Oryza sativa L.) Theor Appl Genet. 1982;63: 373–380. 10.1007/BF00303911 24270875

[21] Hong DL, Leng Y. Genetic analysis of heterosis for number of spikelets per panicle and panicle length of F1 hybrids in japonica rice hybrids. Zhongguo Shuidao Kexue. 2004;18: 255–260.

[22] Yuan LP. Increasing yield potential in rice by exploitation of heterosis In: Virmani SS, editor. Hybrid rice technology: new developments and future prospects. Selected papers from the International Rice Research Conference. Manila: International Rice Research Institute; 1994 pp. 2–6.

[23] Kato T, Takeda K. Association among characters related to yield sink capacity in space-planted rice. Crop Sci. 1996;36: 1135–1139.

[24] Peng S, Cassman KG, Vimani SS, Sheehy J, Khush GS. Yield potential trends of tropical rice since the release of IRS and the challenge of increasing rice yield potential. Crop Sci. 1999;39: 1552–1559.

[25] Wang ET, Wang JJ, Zhu XD, Hao W, Wang L, Li Q, et al Control of rice grain-filling and yield by a gene with a potential signature of domestication. Nat Genet. 2008;40: 1370–1374. 10.1038/ng.220 18820698

[26] Toshiyuki T, Yoshimichi F, Tatsuhiko S, Takeshi H. Time-related mapping of quantitative trait loci controlling grain-filling in rice (Oryza sativa L.). J Exp Bot. 2005;56: 2107–2118. 15983016 10.1093/jxb/eri209

[27] Jia XL, Ye JH, Miao LG, Lin HM, Lin WX. Genetic analysis for grain-filling rate using recombinant inbred lines of (Oryza sativa L.). Zhongguo Nong Xue Tong Bao. 2012;28: 22–26.

[28] Huang JW, Chen JT, Yu WP, Shyur LF, Wang AY, Sun HY, et al Complete structures of three rice sucrose synthase isogenes and differential regulation of their expressions. Biosci Biotechnol Biochem. 1996;60: 233–239. 9063969 10.1271/bbb.60.233

[29] Hirose T, Terao T. A comprehensive expression analysis of the starch synthase gene family in rice (Oryza sativa L.). Planta. 2004;220: 9–16. 15232694 10.1007/s00425-004-1314-6

[30] Okagaki RJ. Nucleotide sequence of a long cDNA from the rice waxy gene. Plant Mol Biol. 1992;19: 513–516. 1377969 10.1007/BF00023402

[31] Akihiro T, Mizuno K, Fujimura T. Gene expression of ADPglucose pyrophosphorylase and starch contents in rice cultured cells are cooperatively regulated by sucrose and ABA. Plant Cell Physiol. 2005;46: 937–946. 15821022 10.1093/pcp/pci101

[32] Agrama HA, Eizenga GC, Yan W. Association mapping of yield and its components in rice cultivars. Mol Breeding. 2007;19: 341–356.

[33] Tian ZX, Qian Q, Liu QQ. Allelic diversities in rice starch biosynthesis lead to a diverse array of rice eating and cooking qualities. Proc Natl Acad Sci U S A. 2009;51: 21760–21765. 10.1073/pnas.0912396106 20018713 PMC2793318

[34] Jin L, Lu Yan, Xiao P, Sun M, Harold C, Bao JS, et al Genetic diversity and population structure of a diverse set of rice germplasm for association mapping. Theor Appl Genet. 2010;121: 475–487. 10.1007/s00122-010-1324-7 20364375

[35] Zhang GH, Gao MG, Zhang GZ, Sun JJ, Jin XM, Jin XM, et al Association analysis of yield traits with molecular markers in Huang-Huai river valley winter wheat region, China. Zuo Wu Xue Bao. 2013;39: 1187–1199.

[36] Jin WD, Cheng BS, Hong DL. Genetic diversity analysis of japonica rice landraces (Oryza sativa L.) in Tai lake region based on SSR markers. Zhong guo nong ye ke xue. 2008;41: 3822–3830.

[37] Cheng BS, Wang ZB, Hong DL. Establishment of SSR finger print map and analysis of genetic similarity among 35 varieties in japonica rice (Oryza sativa L.). Nanjing Nong Ye Da Xue Xue Bao. 2007;30: 1–8.

[38] Temnykh S, Park WD, Ayres N, Cartinhour S, Hauck N, Lipovich L, et al Mapping and genome organization of microsatellite sequences in rice (Oryza sativa L.). Theor Appl Genet. 2000;100: 697–712.

[39] McCouch SR, Teytelman L, Xu YB, Lobos KB, Clare K, Walton M, et al Development and mapping of 2240 new SSR markers for rice (Oryza sativa L.). DNA Res. 2002;9: 199–207. 12597276 10.1093/dnares/9.6.199

[40] Liu EB, Liu Y, Liu XL, Liu QM, Zhao KM, Edzesi W, et al Detecting marker genotypes with elite combining ability for yield traits in parents of hybrid japonica rice. Zhongguo Shuidao Kexue. 2013;27: 473–481.

[41] Wang LQ, Liu WJ, Xu Y, He YQ, Luo LJ, Xing YZ, et al Genetic basis of 17 traits and viscosity parameters characterizing the eating and cooking quality of rice grain. Theor Appl Genet. 2007;115: 463–476. 17593343 10.1007/s00122-007-0580-7

[42] Liu K, Muse SV. PowerMarker: integrated analysis environment for genetic marker data. Bioinformatics. 2005;21: 2128–2129. 15705655 10.1093/bioinformatics/bti282

[43] Bradbury PJ, Zhang Z, Kroon DE, Casstevens TM, Ramdoss Y, Buckler ES, et al TASSEL: software for association mapping of complex traits in diverse samples. Bioinformatics. 2007;23: 2633–2635. 17586829 10.1093/bioinformatics/btm308

[44] Falush D, Stephens M, Pritchard JK. Inference of population structure using multilocus genotype data: linked loci and correlated allele frequencies. Genetics. 2003;164: 1567–1587. 12930761 10.1093/genetics/164.4.1567 PMC1462648

[45] Nei M, Tajima FA, Tateno Y. Accuracy of estimated phylogenetic trees from molecular data. J Mol Evol. 1983;19: 153–170. 6571220 10.1007/BF02300753

[46] Weir BS, Hill WG. Estimating F-statistics. Annu Rev Genet. 2002;36: 721–750. 12359738 10.1146/annurev.genet.36.050802.093940

[47] Excoffier L, Laval G, Schneider S. Arlequin ver. 3.0: an integrated software package for population genetics data analysis. Evol Bioinform Online. 2005;1: 47–50. PMC2658868 19325852

[48] Dang XJ, Tran Thi TG, Dong GS, Wang H, Edzesi W, Hong DL. Genetic diversity and association mapping of seed vigor in rice (Oryza sativa L.). Planta. 2014;239: 1309–1319. 10.1007/s00425-014-2060-z 24668487

[49] Benjamini Y, Hochberg Y. Controlling the false discovery rate: a practical and powerful approach to multiple testing. J R Stat Soc Series B Stat Methodol. 1995;57: 289–300.

[50] Flavio B, Mark E, Sorrells. Association mapping of kernel size and milling quality in wheat (Triticum aestivum L.) cultivars. Genetics. 2006;172: 1165–1177. 16079235 10.1534/genetics.105.044586 PMC1456215

[51] Yoshida S. Fundamentals of rice crop science. Los Baños: International Rice Research Institute; 1981 pp. 72–73.

[52] Yang JC, Zhang JH. Grain-filling problem in super rice. J Exp Bot. 2010; 61: 1–5. 10.1093/jxb/erp348 19959608

[53] Cui D, Xu CY, Tang CF, Yang CG, Yu TQ, A XX, et al Genetic structure and association mapping of cold tolerance in improved japonica rice germplasm at the booting stage. Euphytica. 2013;193: 369–382.

[54] Zhang P, Li J, Li X, Liu XD, Zhao XJ, Lu YG, et al Population structure and genetic diversity in a rice core collection (Oryza sativa L.) investigated with SSR markers. PLOS ONE. 2011;6: e27565 10.1371/journal.pone.0027565 22164211 PMC3229487

[55] Lapitan VC, Brar DS, Abe T, Redoña ED. Assessment of genetic diversity of Philippine rice cultivars carrying good quality traits using SSR markers. Breed Sci. 2007;57: 236–270.

[56] Ma L, Yu XQ, Zhao FS. SSR-based analysis on genetic diversity of rice landraces from Guizhou province, China. Zhongguo Shuidao Kexue. 2010;24: 237–243.

[57] Pritchard JK, Rosenberg NA. Use of unlinked genetic markers to detect population stratification in association studies. Am J Hum Genet. 1999;65: 220–228. 10364535 10.1086/302449 PMC1378093

[58] Garris AJ, Tai TH, Coburn J, Kresovich S, McCouch S. Genetic structure and diversity in Oryza sativa L. Genetics. 2005;169: 1631–1638. 15654106 10.1534/genetics.104.035642 PMC1449546

[59] Flint-Garcia S, Thornsberry J, Buckler ES. Structure of linkage disequilibrium in plants. Annu Rev Plant Biol. 2003;54: 357–374. 14502995 10.1146/annurev.arplant.54.031902.134907

[60] Remington DL, Thornsberry JM, Matsuoka Y, Wilson LM, Whitt SR, Doebley J, et al Structure of linkage disequilibrium and phenotypic associations in the maize genome. Proc Natl Acad Sci U S A. 2001;98: 11479–11484. 11562485 10.1073/pnas.201394398 PMC58755

[61] Risch N, Merikangas K. The future of genetic studies of complex human diseases. Science. 1996;273:1516–1517. 8801636 10.1126/science.273.5281.1516

