# Supplementary material for: Time-Course Association Mapping of the Grain-Filling Rate in Rice (Oryza sativa L.)
Source: PLoS One. 2015 Mar 19;10(3):e0119959. doi: 10.1371/journal.pone.0119959 (PMC4366047; doi:10.1371/journal.pone.0119959)
Supplement: S1 Table — The number 1 and 2 at the top right of accession name indicate the accession belonged to group1 and group2, respectivly. Bold accession names (No.1–58) are the core germplasm collection constructed by Jin et al., doi: 10.3864/j.issn.0578-1752.2008.11.051. The accessions (No.59–95) can be linked on http://www.ricedata.cn/variety/, http://202.127.42.178:4000/countryseed/SpeciesDemand/Default.aspx and http://icscaas.com.cn/sites/ics/ with the accession ID. (DOC) [file pone.0119959.s001.doc]

| **S1 Table. Brown rice weight at five filling stages of the 95 rice germplasm accessions, their geographical origin and ID code that were included in this study.** | | | | | | | | | |
| --- | --- | --- | --- | --- | --- | --- | --- | --- | --- |
| **No.** | **Accession name and ID** | **Origin and**  **geographic coordinates (°E, °N)** | **Year** | **Brown rice weight at 5 filling stages (mg)** | | | | | **Brown rice weight**  **(mg)** |
|  |  |  |  | **7DAF** | **14DAF** | **21DAF** | **28DAF** | **35DAF** |  |
| 1 | **Yazihuang^1^**  (T045) | Jinshan, Shanghai  (121.34, 30.74) | 2011 | 3.0 | 16.0 | 21.2 | 21.5 | 22.3 | 25.0 |
|  |  |  | 2012 | 7.0 | 18.7 | 22.7 | 22.7 | 22.7 | 24.6 |
| 2 | **Hongmangshajing^1^**  (T261) | Kunshan, Jiangsu  (120.98, 31.39) | 2011 | 3.2 | 9.2 | 9.4 | 11.0 | 14.0 | 18.4 |
|  |  |  | 2012 | 3.5 | 8.6 | 15.2 | 15.9 | 16.9 | 18.2 |
| 3 | **Wanhuangdao^1^**  (T815) | Wuxian, Jiangsu  (121.63, 31.26) | 2011 | 7.3 | 17.2 | 20.1 | 20.9 | 20.9 | 21.4 |
|  |  |  | 2012 | 7.0 | 17.5 | 23.5 | 23.8 | 23.8 | 23.8 |
| 4 | **Guozinuo^2^**  (T680) | Jiading, Shanghai  (121.26, 30.74) | 2011 | 4.4 | 12.0 | 20.2 | 18.3 | 20.8 | 23.4 |
|  |  |  | 2012 | 4.2 | 15.9 | 20.6 | 21.9 | 21.9 | 22.0 |
| 5 | **Shuijingbaidao^1^**  (T543) | Wujiang, Jiangsu  (121.34, 31.38) | 2011 | 3.5 | 11.0 | 14.2 | 14.2 | 14.2 | 17.0 |
|  |  |  | 2012 | 1.1 | 12.1 | 18.0 | 18.5 | 18.5 | 18.8 |
| 6 | **Wumangzaodao^1^**  (T442) | Changshu, Jiangsu  (120.75, 31.65) | 2011 | 8.6 | 16.0 | 22.6 | 22.6 | 23.0 | 25.6 |
|  |  |  | 2012 | 8.1 | 18.3 | 24.2 | 24.3 | 24.6 | 24.6 |
| 7 | **Sanbailitou^1^**  (T527) | Kunshan, Jiangsu  (120.98, 31.39) | 2011 | 3.5 | 13.2 | 17.9 | 20.0 | 21.1 | 23.6 |
|  |  |  | 2012 | 1.1 | 10.1 | 15.3 | 20.5 | 24.1 | 24.1 |
| 8 | **Cuyingwanyangdao^1^**  (T585) | Wuxi, Jiangsu  (121.31, 31.49) | 2011 | 4.9 | 14.5 | 21.0 | 23.5 | 24.2 | 27.4 |
|  |  |  | 2012 | 3.6 | 8.1 | 18.2 | 22.8 | 25.3 | 26.8 |
| 9 | **Yanglingdao^1^**  (T632) | Wuxi, Jiangsu  (121.31, 31.49) | 2011 | 4.5 | 12.6 | 18.4 | 18.6 | 20.0 | 23.8 |
|  |  |  | 2012 | 2.6 | 10.1 | 15.4 | 18.7 | 19.2 | 23.8 |
| 10 | **Wanyedao^1^**  (T643) | Wuxian, Jiangsu  (121.63, 31.26) | 2011 | 4.2 | 12.6 | 16.2 | 18.1 | 18.1 | 19.6 |
|  |  |  | 2012 | 2.3 | 9.7 | 17.0 | 19.1 | 19.3 | 19.3 |
| 11 | **Qiaobinghuang^1^**  (T684) | Taicang, Jiangsu  (121.13, 31.46) | 2011 | 6.4 | 13.3 | 14.0 | 15.6 | 16.0 | 17.4 |
|  |  |  | 2012 | 3.0 | 12.6 | 16.4 | 16.4 | 16.9 | 16.9 |
| 12 | **Tieganqing^2^**  (T651) | Wuxian, Jiangsu  (121.63, 31.26) | 2011 | 3.9 | 4.7 | 22.1 | 22.6 | 22.6 | 21.4 |
|  |  |  | 2012 | 1.4 | 9.5 | 18.5 | 19.5 | 19.5 | 20.6 |
| 13 | **Xiaobaiyedao^2^**  (T777) | Wuxi, Jiangsu  (121.31, 31.49) | 2011 | 5.6 | 7.8 | 19.8 | 19.9 | 19.9 | 19.9 |
|  |  |  | 2012 | 2.7 | 12.3 | 17.9 | 18.9 | 19.3 | 19.4 |
| 14 | **Baoxintaihuqing^1^**  (T834) | Wujiang, Jiangsu  (120.65, 31.14) | 2011 | 6.3 | 15.9 | 16.1 | 16.7 | 16.7 | 20.8 |
|  |  |  | 2012 | 5.5 | 18.9 | 20.0 | 20.5 | 20.5 | 20.5 |
| 15 | **Jiangfeng 4^1^**  (T655) | Jiangyin, Jiangsu  (120.29, 31.92) | 2011 | 3.7 | 10.2 | 15.9 | 17.3 | 17.3 | 21.6 |
|  |  |  | 2012 | 4.3 | 6.2 | 16.3 | 17.3 | 17.6 | 21.2 |
| 16 | **Sujing 4^2^**  (T643) | Suzhou, Jiangsu  (120.59, 31.30) | 2011 | 3.0 | 6.6 | 15.7 | 21.8 | 24.3 | 25.2 |
|  |  |  | 2012 | 2.5 | 7.7 | 14.1 | 24.6 | 25.9 | 25.9 |
| 17 | **Aizhongluohanhuang^1^**  (T315) | Wuxi, Jiangsu  (121.31, 31.49) | 2011 | 4.2 | 11.3 | 12.1 | 16.7 | 21.8 | 21.8 |
|  |  |  | 2012 | 4.5 | 9.9 | 19.2 | 21.1 | 22.8 | 22.8 |
| 18 | **Bodao^2^**  (T100) | Wuxi, Jiangsu  (121.31, 31.49) | 2011 | 2.6 | 5.6 | 16.1 | 20.0 | 23.4 | 26.4 |
|  |  |  | 2012 | 3.5 | 8.5 | 14.0 | 22.2 | 24.7 | 25.2 |
| 19 | **Wanmuxiqiu^2^**  (T101) | Taicang, Jiangsu  (121.13, 31.46) | 2011 | 3.2 | 10.0 | 18.8 | 20.0 | 20.2 | 20.8 |
|  |  |  | 2012 | 1.7 | 8.8 | 14.7 | 20.3 | 21.3 | 21.4 |
| 20 | **Huangsanshi^2^**  (T528) | Wujiang, Jiangsu  (120.65, 31.14) | 2011 | 3.5 | 4.6 | 20.5 | 21.9 | 21.9 | 21.9 |
|  |  |  | 2012 | 4.3 | 8.6 | 20.4 | 20.5 | 21.3 | 21.8 |
| 21 | **Erheidao^2^**  (T129) | Wuxi. Jiangsu  (121.31, 31.49) | 2011 | 4.3 | 13.0 | 21.8 | 22.8 | 23.3 | 24.4 |
|  |  |  | 2012 | 5.6 | 13.7 | 22.5 | 23.3 | 24.7 | 25.4 |
| 22 | **Xiaoqingzhong^1^**  (T737) | Changshu, Jiangsu  (120.75, 31.65) | 2011 | 4.6 | 13.9 | 21.0 | 22.0 | 22.2 | 23.6 |
|  |  |  | 2012 | 5.5 | 13.5 | 19.7 | 23.7 | 23.7 | 23.7 |
| 23 | **Zaoguangtou^1^**  (T179) | Wuxi, Jiangsu  (121.31, 31.49) | 2011 | 5.3 | 14.0 | 18.9 | 19.0 | 19.3 | 21.0 |
|  |  |  | 2012 | 4.6 | 13.2 | 14.7 | 20.2 | 20.6 | 21.2 |
| 24 | **Xiaoluohanhuang^2^**  (T325) | Changshu, Jiangsu  (120.75, 31.65) | 2011 | 3.8 | 11.3 | 19.8 | 19.9 | 20.8 | 21.8 |
|  |  |  | 2012 | 5.8 | 15.1 | 17.0 | 18.1 | 19.7 | 21.6 |
| 25 | **Suzhouqing^1^**  (T167) | Jiangyin, Jiangsu  (120.29, 31.92) | 2011 | 2.0 | 13.8 | 15.3 | 18.5 | 18.5 | 21.4 |
|  |  |  | 2012 | 1.9 | 9.1 | 15.7 | 19.1 | 20.0 | 21.8 |
| 26 | **Wanluli^1^**  (T331) | Jiangyin, Jiangsu  (120.29, 31.92) | 2011 | 5.1 | 14.2 | 20.4 | 20.6 | 20.9 | 22.6 |
|  |  |  | 2012 | 3.8 | 9.1 | 16.4 | 22.8 | 22.8 | 22.8 |
| 27 | **Wanbaguo^1^**  (T357) | Jiangyin, Jiangsu  (120.29, 31.92) | 2011 | 3.8 | 14.6 | 21.3 | 22.3 | 25.1 | 26.0 |
|  |  |  | 2012 | 5.3 | 9.3 | 15.2 | 23.6 | 23.8 | 25.6 |
| 28 | **Ebusinuodao^2^**  (T386) | Wuxi, Jiangsu  (121.31, 31.49) | 2011 | 3.3 | 10.1 | 17.4 | 18.2 | 18.2 | 18.6 |
|  |  |  | 2012 | 1.3 | 2.0 | 18.3 | 18.0 | 18.8 | 19.6 |
| 29 | **Laodeigu^1^**  (T397) | Wujiang, Jiangsu  (120.65, 31.14) | 2011 | 3.1 | 9.1 | 14.6 | 18.4 | 19.5 | 23.0 |
|  |  |  | 2012 | 1.8 | 8.3 | 14.9 | 19.0 | 20.7 | 22.8 |
| 30 | **Yefenghuang^1^**  (T402) | Wujiang, Jiangsu  (120.65, 31.14) | 2011 | 4.1 | 11.2 | 18.0 | 20.9 | 22.0 | 22.0 |
|  |  |  | 2012 | 4.7 | 14.3 | 20.3 | 22.7 | 22.7 | 22.7 |
| 31 | **Chenjiazhong^1^**  (T554) | Kunshan, Jiangsu  (120.98, 31.39) | 2011 | 4.7 | 16.0 | 18.6 | 20.3 | 20.3 | 21.8 |
|  |  |  | 2012 | 4.8 | 10.3 | 17.3 | 21.2 | 21.5 | 21.5 |
| 32 | **Zaoheitouhong^1^**  (T473) | Wujiang, Jiangsu  (120.65, 31.14) | 2011 | 2.1 | 8.5 | 13.2 | 16.7 | 17.0 | 22.6 |
|  |  |  | 2012 | 1.9 | 8.7 | 14.4 | 19.9 | 19.9 | 21.8 |
| 33 | **Luohanghuang^2^**  (T560) | Jiangyin, Jiangsu  (120.29, 31.92) | 2011 | 3.0 | 7.6 | 15.5 | 22.2 | 23.8 | 25.4 |
|  |  |  | 2012 | 1.6 | 8.0 | 11.1 | 23.1 | 24.9 | 26.6 |
| 34 | **Longgouzhong^1^**  (T580) | Qingpu, Shanghai  (121.13, 31.15) | 2011 | 4.2 | 16.2 | 22.0 | 22.7 | 22.7 | 27.4 |
|  |  |  | 2012 | 1.8 | 8.6 | 14.4 | 19.8 | 23.7 | 26.4 |
| 35 | **Shiluqing^2^**  (T652) | Kunshan, Jiangsu  (120.98, 31.39) | 2011 | 4.5 | 7.1 | 14.8 | 17.1 | 18.1 | 23.2 |
|  |  |  | 2012 | 2.9 | 7.7 | 11.1 | 18.2 | 19.7 | 21.6 |
| 36 | **Ligengqing^2^**  (T656) | Yixing, Jiangsu  (119.82, 31.34) | 2011 | 4.8 | 8.6 | 17.4 | 18.6 | 18.6 | 21.4 |
|  |  |  | 2012 | 1.6 | 7.3 | 12.7 | 18.4 | 19.1 | 21.2 |
| 37 | **Zaoheitouhong2^1^**  (T474) | Wujiang, Jiangsu  (120.65, 31.14) | 2011 | 3.7 | 11.4 | 17.7 | 15.8 | 21.7 | 22.4 |
|  |  |  | 2012 | 1.7 | 9.8 | 13.5 | 21.9 | 21.9 | 22.2 |
| 38 | **Laolaihong^2^**  (T689) | Wuxian, Jiangsu  (121.63, 31.26) | 2011 | 4.6 | 9.0 | 16.1 | 20.5 | 20.9 | 25.2 |
|  |  |  | 2012 | 3.9 | 10.8 | 16.6 | 19.4 | 23.0 | 26.2 |
| 39 | **Erlibie^1^**  (T701) | Wuxian, Jiangsu  (121.63, 31.26) | 2011 | 4.0 | 12.9 | 17.6 | 19.0 | 20.1 | 25.2 |
|  |  |  | 2012 | 5.2 | 15.5 | 20.9 | 21.7 | 21.7 | 24.2 |
| 40 | **Jinguhuang^1^**  (T841) | Kunshan, Jiangsu  (120.98, 31.39) | 2011 | 2.3 | 8.6 | 14.1 | 17.9 | 20.7 | 21.2 |
|  |  |  | 2012 | 3.3 | 10.5 | 16.3 | 20.7 | 20.8 | 20.8 |
| 41 | **Cuganhuangdao^1^**  (T718) | Wujiang, Jiangsu  (120.65, 31.14) | 2011 | 3.2 | 15.7 | 16.4 | 18.3 | 20.4 | 24.0 |
|  |  |  | 2012 | 1.1 | 8.3 | 12.8 | 17.4 | 20.8 | 23.8 |
| 42 | **Zaoshirihuangdao^2^**  (T728) | Wuxian, Jiangsu  (121.63, 31.26) | 2011 | 3.4 | 3.8 | 11.3 | 18.0 | 19.2 | 22.6 |
|  |  |  | 2012 | 1.1 | 9.5 | 14.2 | 16.8 | 20.2 | 22.8 |
| 43 | **Shengtangqing^1^**  (T759) | Changshu, Jiangsu  (120.75, 31.65) | 2011 | 3.6 | 9.8 | 12.2 | 18.0 | 21.3 | 24.6 |
|  |  |  | 2012 | 2.6 | 7.5 | 11.6 | 16.5 | 20.8 | 23.8 |
| 44 | **Xiaomandao^1^**  (T750) | Wuxian, Jiangsu  (121.63, 31.26) | 2011 | 3.2 | 13.5 | 18.9 | 20.7 | 23.7 | 26.2 |
|  |  |  | 2012 | 1.5 | 8.8 | 13.7 | 20.8 | 22.2 | 25.0 |
| 45 | **Shengtangqing2^1^**  (T206) | Changshu, Jiangsu  (120.75, 31.65) | 2011 | 3.2 | 12.1 | 16.3 | 16.9 | 17.0 | 17.2 |
|  |  |  | 2012 | 5.8 | 16.2 | 16.3 | 16.5 | 16.6 | 16.8 |
| 46 | **Wanmandao^2^**  (T772) | Wujiang, Jiangsu  (120.65, 31.14) | 2011 | 4.4 | 6.2 | 14.5 | 17.7 | 18.1 | 23.2 |
|  |  |  | 2012 | 4.2 | 6.3 | 14.7 | 17.9 | 18.9 | 23.2 |
| 47 | **Nantouzhong^1^**  (T600) | Kunshan, Jiangsu  (120.98, 31.39) | 2011 | 8.1 | 15.4 | 19.7 | 21.7 | 21.7 | 23.8 |
|  |  |  | 2012 | 5.7 | 14.3 | 20.9 | 23.1 | 23.9 | 24.0 |
| 48 | **Daniaodao^1^**  (T453) | Changshu, Jiangsu  (120.75, 31.65) | 2011 | 4.5 | 14.5 | 15.5 | 22.6 | 24.6 | 28.2 |
|  |  |  | 2012 | 5.1 | 12.3 | 15.5 | 24.7 | 24.9 | 28.8 |
| 49 | **Kongqueqing^1^**  (T833) | Kunshan, Jiangsu  (120.98, 31.39) | 2011 | 2.9 | 13.1 | 21.0 | 19.6 | 21.1 | 22.2 |
|  |  |  | 2012 | 1.9 | 4.9 | 17.4 | 19.2 | 22.7 | 22.7 |
| 50 | **Kaiqing^1^**  (T232) | Wuxian, Jiangsu  (121.63, 31.26) | 2011 | 4.0 | 8.7 | 11.6 | 14.8 | 16.9 | 16.9 |
|  |  |  | 2012 | 2.6 | 9.1 | 17.7 | 17.7 | 17.7 | 17.8 |
| 51 | **Manyedao^1^**  (T513) | Kunshan, Jiangsu  (120.98, 31.39) | 2011 | 2.9 | 10.9 | 16.2 | 18.7 | 21.0 | 22.0 |
|  |  |  | 2012 | 1.9 | 10.8 | 16.1 | 20.6 | 20.6 | 22.8 |
| 52 | **Baikenuo^1^**  (T354) | Wuxian, Jiangsu  (121.63, 31.26) | 2011 | 5.0 | 14.2 | 15.2 | 17.3 | 18.1 | 19.6 |
|  |  |  | 2012 | 6.3 | 18.9 | 19.4 | 19.4 | 19.9 | 19.9 |
| 53 | **Baimangnuo^1^**  (T033) | Wujiang, Jiangsu  (120.65, 31.14) | 2011 | 3.5 | 12.2 | 13.7 | 16.6 | 17.7 | 19.2 |
|  |  |  | 2012 | 3.2 | 11.7 | 17.5 | 17.5 | 17.5 | 19.8 |
| 54 | **Xiangzhunuo^1^**  (T452) | Changshu, Jiangsu  (120.75, 31.65) | 2011 | 3.1 | 10.3 | 17.6 | 17.9 | 18.9 | 19.6 |
|  |  |  | 2012 | 2.5 | 10.1 | 15.7 | 16.8 | 18.7 | 20.6 |
| 55 | **Yaxuenuo^2^**  (T480) | Wuxian, Jiangsu  (121.63, 31.26) | 2011 | 3.7 | 9.3 | 17.2 | 18.7 | 19.3 | 19.4 |
|  |  |  | 2012 | 2.1 | 8.7 | 16.1 | 19.5 | 20.1 | 20.1 |
| 56 | **Xianhui429^1^**  (T573) | Nanjing, Jiangsu  (118.64, 32.07) | 2011 | 7.9 | 12.2 | 17.4 | 17.3 | 17.8 | 18.8 |
|  |  |  | 2012 | 5.2 | 17.9 | 18.0 | 18.1 | 18.8 | 19.2 |
| 57 | **Zijianxian3^1^**  (T504) | Nanjing, Jiangsu  (118.64, 32.07) | 2011 | 6.2 | 14.0 | 16.6 | 16.6 | 17.8 | 18.2 |
|  |  |  | 2012 | 4.4 | 13.3 | 16.4 | 16.9 | 18.2 | 18.2 |
| 58 | **Huangsandannuo^1^**  (T106) | Wuxian, Jiangsu  (121.63, 31.26) | 2011 | 4.6 | 11.8 | 13.8 | 14.3 | 14.3 | 16.4 |
|  |  |  | 2012 | 2.7 | 16.4 | 16.5 | 16.5 | 16.8 | 16.8 |
| 59 | Jia 159^2^  (Zheshendao2003008) | Jiaxing, Zhejiang  (120.76, 30.75) | 2011 | 3.2 | 4.70 | 12.4 | 14.4 | 22.6 | 24.0 |
|  |  |  | 2012 | 1.9 | 7.40 | 13.2 | 14.5 | 21.5 | 24.2 |
| 60 | Sidao 10^1^  (ZD-05529) | Siyang, Jiangsu  (118.70, 33.72) | 2011 | 5.1 | 9.10 | 14.4 | 18.9 | 21.4 | 24.1 |
|  |  |  | 2012 | 5.5 | 15.7 | 22.0 | 22.5 | 22.5 | 23.0 |
| 61 | Wuqiang^2^  (ZD-05545) | Wujin, Jiangsu  (119.94, 31.70) | 2011 | 3.2 | 8.8 | 17.7 | 20.0 | 22.3 | 25.2 |
|  |  |  | 2012 | 3.3 | 7.2 | 14.7 | 20.3 | 22.1 | 25.0 |
| 62 | Wuyujing 3^2^  (Suzhongshenzidi156) | Wujin, Jiangsu  (119.94, 31.70) | 2011 | 4.3 | 8.4 | 17.1 | 19.4 | 20.4 | 22.0 |
|  |  |  | 2012 | 3.9 | 15.0 | 17.7 | 21.1 | 22.7 | 23.0 |
| 63 | Xiushui 04^1^  (GS01009-1990) | Jiaxing,Zhejiang  (120.76, 30.75) | 2011 | 2.9 | 9.3 | 14.4 | 18.9 | 22.5 | 26.0 |
|  |  |  | 2012 | 1.8 | 9.1 | 12.6 | 20.2 | 22.1 | 25.6 |
| 64 | Zhendao 88^1^  (Suzhongshenzidi 265) | Zhenjiang, Jiangsu  (119.43, 32.19) | 2011 | 2.7 | 10.9 | 13.3 | 18.3 | 22.5 | 24.4 |
|  |  |  | 2012 | 2.6 | 10.5 | 16.4 | 20.7 | 23.1 | 24.8 |
| 65 | Zhendao 6^1^  (Guoshendao990009) | Zhenjiang, Jiangsu  (119.43, 32.19) | 2011 | 3.1 | 10.1 | 15.4 | 18.6 | 23.6 | 26.4 |
|  |  |  | 2012 | 3.9 | 10.1 | 17.1 | 20.8 | 25.1 | 26.2 |
| 66 | Taijing9xuan^1^  (ZD-05548) | Taibei,Taiwan  (121.49, 25.05) | 2011 | 2.4 | 11.1 | 15.8 | 18.9 | 22 | 23.8 |
|  |  |  | 2012 | 5.3 | 16.3 | 22.1 | 23 | 23.2 | 23.6 |
| 67 | ACTaijing16xuandi^1^  (ZD-05549) | Taibei,Taiwan  (121.49, 25.05) | 2011 | 3.1 | 8.9 | 13.4 | 17.1 | 20.2 | 22.6 |
|  |  |  | 2012 | 3.9 | 13.7 | 18.4 | 21.5 | 21.6 | 21.8 |
| 68 | Taijing16xuanzi^2^  (ZD-05550) | Taibei,Taiwan  (121.49, 25.05) | 2011 | 3.7 | 8.8 | 16.3 | 18.7 | 19.1 | 21.6 |
|  |  |  | 2012 | 4.8 | 11.8 | 17.9 | 21.9 | 21.9 | 21.9 |
| 69 | Diantun502xuanzao^1^  (09-02598) | Kunming,Yunnan  (102.83, 24.88) | 2011 | 2.9 | 15.8 | 17.6 | 21.5 | 22.3 | 22.9 |
|  |  |  | 2012 | 2.7 | 11.9 | 19.9 | 21.2 | 22.2 | 22.2 |
| 70 | H35( 6435)^2^  (ZD-05552) | Nanjing, Jiangsu  (118.64, 32.07) | 2011 | 2.9 | 10.2 | 17.8 | 19.1 | 23.1 | 29.2 |
|  |  |  | 2012 | 1.8 | 8.9 | 16.7 | 24.9 | 24.9 | 29.0 |
| 71 | H37( 6427)^2^  (ZD-05553) | Nanjing, Jiangsu  (118.64, 32.07) | 2011 | 3.2 | 7.3 | 16.0 | 17.1 | 18.3 | 19.2 |
|  |  |  | 2012 | 1.9 | 9.0 | 15.6 | 16.9 | 17.7 | 18.1 |
| 72 | Jingnuo(zijian)^2^  (ZD-05556) | Nanjing, Jiangsu  (118.64, 32.07) | 2011 | 4.7 | 8.9 | 15.4 | 16.7 | 18.6 | 21.0 |
|  |  |  | 2012 | 3.6 | 8.8 | 14.3 | 19.1 | 19.5 | 20.6 |
| 73 | Nannongjing62401^1^  (ZD-05557) | Nanjing, Jiangsu  (118.64, 32.07) | 2011 | 8.2 | 17.2 | 18.9 | 19.8 | 19.8 | 19.8 |
|  |  |  | 2012 | 7.2 | 18.0 | 18.3 | 18.6 | 18.6 | 18.6 |
| 74 | Tongjing109^1^  (Suzhongshenzidi 296) | Nantong, Jiangsu  (120.89, 31.98) | 2011 | 4.1 | 14.2 | 20.1 | 22.1 | 23.7 | 24.8 |
|  |  |  | 2012 | 6.1 | 20.2 | 20.7 | 23.9 | 23.9 | 25.2 |
| 75 | Yangdao 6^1^  (Guoshendao2001002) | Yangzhou, Jiangsu  (119.41, 32.39) | 2011 | 3.7 | 16.3 | 16.6 | 17.0 | 17.4 | 18.1 |
|  |  |  | 2012 | 7.1 | 16.3 | 17.3 | 17.5 | 18.4 | 18.8 |
| 76 | Ninjing 1^2^  (Sushendao200417) | Nanjing, Jiangsu  (118.64, 32.07) | 2011 | 4.3 | 8.7 | 19.3 | 23.0 | 24.1 | 27.4 |
|  |  |  | 2012 | 2.6 | 4.8 | 15.7 | 21.1 | 23.9 | 26.6 |
| 77 | Wujing 15^2^  (Sushendao200418) | Wujin, Jiangsu  (119.94, 31.70) | 2011 | 3.4 | 12.0 | 20.7 | 22.0 | 22.0 | 26.2 |
|  |  |  | 2012 | 2.7 | 11.1 | 13.1 | 18.9 | 23.4 | 25.4 |
| 78 | Wuxiangjing 14^1^  (Sushendao200315) | Wujin, Jiangsu  (119.94, 31.70) | 2011 | 2.9 | 13.3 | 17.5 | 22.2 | 22.9 | 25.2 |
|  |  |  | 2012 | 3.1 | 9.0 | 14.1 | 22.7 | 23.1 | 24.1 |
| 79 | Xudao 3^2^  (Sushendao200306) | Xuzhou, Jiangsu  (117.28, 34.21) | 2011 | 4.2 | 6.8 | 15.1 | 18.1 | 19.2 | 20.0 |
|  |  |  | 2012 | 2.7 | 7.3 | 14.3 | 17.1 | 19.1 | 19.8 |
| 80 | Nannongjing 003^2^  (ZD-05572) | Nanjing, Jiangsu  (118.64, 32.07) | 2011 | 4.3 | 10.2 | 17.4 | 19.2 | 20.5 | 25.2 |
|  |  |  | 2012 | 4.3 | 11.4 | 19.1 | 19.5 | 20.0 | 25.2 |
| 81 | Nannongjing 005^1^  (ZD-05574) | Nanjing, Jiangsu  (118.64, 32.07) | 2011 | 3.4 | 12.3 | 15.4 | 16.2 | 18.3 | 20.2 |
|  |  |  | 2012 | 2.5 | 8.8 | 14.1 | 17.1 | 20.1 | 20.1 |
| 82 | 5jing20^2^  (ZD-05575) | Nanjing, Jiangsu  (118.64, 32.07) | 2011 | 2.1 | 7.0 | 18.5 | 18.6 | 20.0 | 20.8 |
|  |  |  | 2012 | 4.6 | 12.0 | 18.7 | 21.2 | 22.7 | 22.7 |
| 83 | 5jing15^2^  (ZD-05576) | Nanjing, Jiangsu  (118.64, 32.07) | 2011 | 3.9 | 10.9 | 18.4 | 22.4 | 22.4 | 23.1 |
|  |  |  | 2012 | 2.9 | 10.0 | 19.6 | 23.5 | 24.9 | 24.9 |
| 84 | Molingjing^1^  (ZD-05577) | Jiangning, Jiangsu  (118.84, 31.96) | 2011 | 5.0 | 16.1 | 21.1 | 25.8 | 25.8 | 26.0 |
|  |  |  | 2012 | 3.2 | 11.1 | 17.5 | 24.5 | 25.1 | 25.8 |
| 85 | 5jing03^2^  (ZD-05578) | Nanjing, Jiangsu  (118.64, 32.07) | 2011 | 2.5 | 9.2 | 16.4 | 19.9 | 22.9 | 24.9 |
|  |  |  | 2012 | 2.6 | 9.3 | 18.5 | 21.5 | 23.5 | 24.4 |
| 86 | 5jing68^1^  (ZD-05579) | Nanjing, Jiangsu  (118.64, 32.07) | 2011 | 4.3 | 12.6 | 19.6 | 20.8 | 22.1 | 26.6 |
|  |  |  | 2012 | 3.1 | 16.1 | 22.2 | 24.9 | 25.1 | 26.0 |
| 87 | Xudao 4^1^  (ZD-05646) | Xuzhou, Jiangsu  (117.28, 34.21) | 2011 | 2.5 | 11.7 | 15.4 | 20.2 | 23.5 | 23.8 |
|  |  |  | 2012 | 5.7 | 11.9 | 23.2 | 23.2 | 24.1 | 25.6 |
| 88 | Xudao 5^1^  (Guoshendao2006059) | Xuzhou, Jiangsu  (117.28, 34.21) | 2011 | 3.9 | 10.0 | 14.5 | 19.2 | 22.6 | 25.2 |
|  |  |  | 2012 | 4.8 | 12.5 | 17.1 | 19.5 | 21.9 | 25.6 |
| 89 | Huaidao 9^1^  (ZD-05655) | Xuzhou, Jiangsu  (117.28, 34.21) | 2011 | 3.0 | 12.9 | 20.9 | 21.8 | 22.0 | 26.0 |
|  |  |  | 2012 | 4.7 | 13.5 | 24.7 | 25.3 | 25.6 | 26.2 |
| 90 | Yandao 6^1^  (Sushendao200205) | Yancheng, Jiangsu  (120.16, 33.35) | 2011 | 5.0 | 12.0 | 17.0 | 20.7 | 21.0 | 22.6 |
|  |  |  | 2012 | 4.5 | 13.9 | 21.9 | 22.7 | 22.7 | 22.7 |
| 91 | Yangguang 200^1^  (Guoshendao2008043) | Xuzhou, Jiangsu  (117.28, 34.21) | 2011 | 4.1 | 14.4 | 18.8 | 20.1 | 20.5 | 24.6 |
|  |  |  | 2012 | 4.3 | 15.7 | 20.7 | 22.2 | 22.8 | 25.0 |
| 92 | Lianjing 2^1^  (Guoshendao990021) | Xuzhou, Jiangsu  (117.28, 34.21) | 2011 | 3.2 | 5.7 | 16.5 | 17.4 | 20.5 | 22.6 |
|  |  |  | 2012 | 3.7 | 9.7 | 19.5 | 21.3 | 22.0 | 23.0 |
| 93 | Xiushui79^1^  (Guoshendao2008021) | Jiaxing, Zhejiang  (120.76, 30.75) | 2011 | 4.7 | 10.1 | 14.3 | 14.3 | 21.2 | 23.4 |
|  |  |  | 2012 | 4.0 | 12.5 | 18.5 | 18.8 | 22.5 | 23.0 |
| 94 | C bao^2^  (92010109) | Hefei,Anhui  (117.22, 31.82) | 2011 | 2.5 | 9.5 | 19.0 | 22.0 | 22.0 | 22.8 |
|  |  |  | 2012 | 2.2 | 8.5 | 18.4 | 21.0 | 24.2 | 24.2 |
| 95 | Zhen9424^2^  (ZD-05658) | Zhenjiang, Jiangsu  (119.43, 32.19) | 2011 | 5.5 | 14.2 | 18.2 | 19.0 | 22.9 | 22.9 |
|  |  |  | 2012 | 4.7 | 15.1 | 19.5 | 19.8 | 20.5 | 20.6 |
| The number 1 and 2 at the top right of accession name indicate the accession belonged to group1 and group2, respectivly. Bold accession names (No.1-58) are the core germplasm collection constructed by Jin et al., doi: 10.3864/j.issn.0578-1752.2008.11.051. The accessions (No.59-95) can be linked on <http://www.ricedata.cn/variety/> , <http://202.127.42.178:4000/countryseed/SpeciesDemand/Default.aspx> and <http://icscaas.com.cn/sites/ics/> with the accession ID. | | | | | | | | | |
