# Supplementary material for: Time-Course Association Mapping of the Grain-Filling Rate in Rice (Oryza sativa L.)
Source: PLoS One. 2015 Mar 19;10(3):e0119959. doi: 10.1371/journal.pone.0119959 (PMC4366047; doi:10.1371/journal.pone.0119959)
Supplement: S2 Table — (DOC) [file pone.0119959.s002.doc]

| **S2 Table. Summary statistics for the 263 SSR markers used in the present study** | | | | | | | | | | | |
| --- | --- | --- | --- | --- | --- | --- | --- | --- | --- | --- | --- |
| **Locus** | **Chr. No.** | **Position**  **(cM)** | **Allele number** | **Genetic diversity** | **PIC** | **Locus** | **Chr. No.** | **Position**  **(cM)** | **Allele number** | **Genetic diversity** | **PIC** |
| RM84 | 1 | 18.8 | 5 | 0.5392 | 0.4856 | RM136 | 6 | 53.0 | 7 | 0.7716 | 0.7393 |
| RM1-003 | 1 | 19.9 | 8 | 0.7419 | 0.7065 | RM3330 | 6 | 61.6 | 5 | 0.5970 | 0.5176 |
| RM283 | 1 | 25.4 | 4 | 0.5190 | 0.4497 | RM3187 | 6 | 73.2 | 6 | 0.5207 | 0.4932 |
| RM3453 | 1 | 25.4 | 8 | 0.7570 | 0.7195 | RM7579 | 6 | 84.5 | 6 | 0.6307 | 0.5705 |
| RM1 | 1 | 29.7 | 10 | 0.7725 | 0.7385 | RM8239 | 6 | 91.9 | 3 | 0.4805 | 0.3749 |
| RM259 | 1 | 38.8 | 6 | 0.2633 | 0.2491 | RM454 | 6 | 99.3 | 5 | 0.5175 | 0.4288 |
| RM583 | 1 | 43.2 | 6 | 0.5722 | 0.5155 | RM7309 | 6 | 100.3 | 12 | 0.8091 | 0.7917 |
| RM490 | 1 | 51.0 | 6 | 0.2305 | 0.2210 | RM528 | 6 | 100.8 | 5 | 0.1968 | 0.1925 |
| RM8095 | 1 | 60.6 | 4 | 0.2433 | 0.2260 | RM3138 | 6 | 110.6 | 7 | 0.6045 | 0.5713 |
| RM140 | 1 | 65.4 | 3 | 0.1201 | 0.1162 | RM162 | 6 | 114.9 | 6 | 0.6593 | 0.6219 |
| RM562 | 1 | 78.4 | 8 | 0.7597 | 0.7245 | RM6811 | 6 | 115.6 | 13 | 0.8470 | 0.8297 |
| RM9 | 1 | 92.4 | 10 | 0.7143 | 0.6901 | RM345 | 6 | 123.9 | 4 | 0.1398 | 0.1362 |
| RM129 | 1 | 93.0 | 2 | 0.0997 | 0.0948 | RM5753 | 6 | 124.4 | 12 | 0.8332 | 0.8157 |
| RM5 | 1 | 98.5 | 6 | 0.6156 | 0.5617 | RM295 | 7 | 0.0 | 4 | 0.5981 | 0.5173 |
| RM1231 | 1 | 123.2 | 9 | 0.7898 | 0.7612 | RM125 | 7 | 24.8 | 4 | 0.5086 | 0.4239 |
| RM128 | 1 | 126.5 | 10 | 0.8490 | 0.8314 | RM180 | 7 | 30.1 | 4 | 0.5786 | 0.5341 |
| RM297 | 1 | 132.0 | 8 | 0.6838 | 0.6327 | RM542 | 7 | 34.7 | 7 | 0.7224 | 0.6751 |
| RM246 | 1 | 134.6 | 7 | 0.6057 | 0.5268 | RM8263 | 7 | 35.7 | 6 | 0.7863 | 0.7535 |
| RM212 | 1 | 135.8 | 4 | 0.1768 | 0.1708 | RM418 | 7 | 42.1 | 6 | 0.6755 | 0.6174 |
| RM5389 | 1 | 142.4 | 8 | 0.6970 | 0.6455 | RM346 | 7 | 47.0 | 4 | 0.5746 | 0.4823 |
| RM486 | 1 | 153.5 | 7 | 0.6741 | 0.6197 | RM2530 | 7 | 53.4 | 6 | 0.7364 | 0.6948 |
| RM265 | 1 | 155.9 | 5 | 0.2130 | 0.2048 | RM336 | 7 | 61.0 | 6 | 0.7119 | 0.6621 |
| RM3482 | 1 | 157.6 | 8 | 0.6012 | 0.5741 | RM5380 | 7 | 67.0 | 6 | 0.7258 | 0.6782 |
| RM6831 | 1 | 181.8 | 4 | 0.5308 | 0.4205 | RM6011 | 7 | 73.2 | 6 | 0.7130 | 0.6661 |
| RM14 | 1 | 194.0 | 6 | 0.5186 | 0.4309 | RM505 | 7 | 78.6 | 3 | 0.2909 | 0.2544 |
| RM5340 | 2 | 36.3 | 8 | 0.7612 | 0.7222 | RM3589 | 7 | 89.8 | 6 | 0.7083 | 0.6624 |
| RM7288 | 2 | 42.4 | 4 | 0.0625 | 0.0618 | RM11 | 7 | 93.8 | 6 | 0.5669 | 0.5003 |
| RM5356 | 2 | 43.3 | 6 | 0.5005 | 0.4547 | RM234 | 7 | 93.9 | 5 | 0.5693 | 0.4936 |
| RM1358 | 2 | 48.1 | 5 | 0.5837 | 0.5262 | RM134 | 7 | 99.6 | 5 | 0.6150 | 0.5372 |
| RM1313 | 2 | 51.1 | 7 | 0.4089 | 0.3895 | RM1306 | 7 | 116.1 | 8 | 0.7453 | 0.7045 |
| RM324 | 2 | 51.1 | 6 | 0.2123 | 0.2031 | RM82 | 7 | 128.9 | 4 | 0.3984 | 0.3669 |
| RM327 | 2 | 51.9 | 3 | 0.0423 | 0.0419 | RM506 | 8 | 0.0 | 5 | 0.6283 | 0.5572 |
| RM301 | 2 | 53.5 | 6 | 0.6019 | 0.5251 | RM1019 | 8 | 0.5 | 8 | 0.7231 | 0.6776 |
| RM300 | 2 | 54.6 | 6 | 0.5994 | 0.5196 | RM152 | 8 | 9.4 | 4 | 0.6322 | 0.5646 |
| RM262 | 2 | 70.2 | 7 | 0.5771 | 0.4879 | RM1235 | 8 | 12.8 | 5 | 0.4359 | 0.3789 |
| RM5427 | 2 | 84.6 | 5 | 0.4270 | 0.3940 | RM6863 | 8 | 16.4 | 6 | 0.5614 | 0.4649 |
| RM3688 | 2 | 88.2 | 7 | 0.7499 | 0.7079 | RM4085 | 8 | 35.7 | 4 | 0.6688 | 0.6116 |
| RM183 | 2 | 93.5 | 5 | 0.5511 | 0.4511 | RM544 | 8 | 38.5 | 3 | 0.5104 | 0.3904 |
| RM5804 | 2 | 98.2 | 6 | 0.5046 | 0.3971 | RM8243 | 8 | 50.8 | 4 | 0.5104 | 0.3904 |
| RM106 | 2 | 101.5 | 9 | 0.7852 | 0.7540 | RM25 | 8 | 52.2 | 6 | 0.6082 | 0.5336 |
| RM6361 | 2 | 102.9 | 5 | 0.5039 | 0.4059 | RM331 | 8 | 59.0 | 5 | 0.2314 | 0.2232 |
| RM573 | 2 | 118.1 | 13 | 0.8481 | 0.8316 | RM72 | 8 | 60.9 | 4 | 0.6775 | 0.6197 |
| RM450 | 2 | 122.8 | 8 | 0.7293 | 0.6938 | RM6215 | 8 | 66.8 | 6 | 0.7033 | 0.6542 |
| RM7598 | 2 | 126.4 | 5 | 0.4784 | 0.3997 | RM7556 | 8 | 86.7 | 5 | 0.5811 | 0.4939 |
| RM263 | 2 | 127.5 | 4 | 0.6295 | 0.5590 | RM6976 | 8 | 92.2 | 6 | 0.5862 | 0.5126 |
| RM112 | 2 | 137.5 | 6 | 0.5201 | 0.4053 | RM80 | 8 | 103.7 | 7 | 0.6152 | 0.5850 |
| RM525 | 2 | 143.7 | 4 | 0.3887 | 0.3639 | RM502 | 8 | 109.3 | 4 | 0.6292 | 0.5603 |
| RM213 | 2 | 150.5 | 6 | 0.3762 | 0.3326 | RM3754 | 8 | 112.6 | 5 | 0.6421 | 0.5866 |
| RM208 | 2 | 154.1 | 5 | 0.4948 | 0.4474 | RM6948 | 8 | 114.4 | 4 | 0.5315 | 0.4443 |
| RM3850 | 2 | 156.3 | 6 | 0.7118 | 0.6671 | RM433 | 8 | 116.0 | 2 | 0.0208 | 0.0206 |
| RM498 | 2 | 156.3 | 14 | 0.8317 | 0.8142 | RM281 | 8 | 128.1 | 4 | 0.5404 | 0.4347 |
| RM48 | 2 | 191.2 | 4 | 0.3982 | 0.3343 | RM264 | 8 | 138.2 | 3 | 0.4635 | 0.3658 |
| RM266 | 2 | 192.2 | 7 | 0.5086 | 0.4864 | RM1328 | 9 | 0.0 | 6 | 0.7293 | 0.6834 |
| RM535 | 2 | 195.7 | 5 | 0.6023 | 0.5231 | RM8206 | 9 | 3.2 | 6 | 0.7612 | 0.7200 |
| RM132 | 3 | 3.9 | 5 | 0.5624 | 0.4824 | RM524 | 9 | 42.5 | 5 | 0.6815 | 0.6234 |
| RM1332 | 3 | 11.5 | 5 | 0.5305 | 0.4204 | RM3912 | 9 | 46.3 | 8 | 0.7379 | 0.6927 |
| RM5849 | 3 | 18.4 | 12 | 0.7129 | 0.6827 | RM566 | 9 | 50.7 | 4 | 0.5315 | 0.4443 |
| RM489 | 3 | 20.3 | 7 | 0.6143 | 0.5660 | RM434 | 9 | 57.7 | 3 | 0.5104 | 0.3904 |
| RM545 | 3 | 24.7 | 4 | 0.1921 | 0.1804 | RM3600 | 9 | 62.7 | 8 | 0.7264 | 0.6877 |
| RM5480 | 3 | 25.9 | 7 | 0.7001 | 0.6457 | RM24481 | 9 | 63.0 | 4 | 0.5830 | 0.4932 |
| RM3467 | 3 | 28.2 | 11 | 0.6429 | 0.6075 | RM3533 | 9 | 65.1 | 5 | 0.6026 | 0.5231 |
| RM3766 | 3 | 34.8 | 9 | 0.6091 | 0.5746 | RM6570 | 9 | 68.2 | 3 | 0.4715 | 0.3700 |
| RM7 | 3 | 36.9 | 7 | 0.5901 | 0.5420 | RM410 | 9 | 79.3 | 5 | 0.6193 | 0.5451 |
| RM5639 | 3 | 39.8 | 7 | 0.4672 | 0.4277 | RM257 | 9 | 79.7 | 9 | 0.7168 | 0.6816 |
| RM7197 | 3 | 44.4 | 4 | 0.2017 | 0.1888 | RM201 | 9 | 81.2 | 6 | 0.6373 | 0.5667 |
| RM7345 | 3 | 48.8 | 5 | 0.2128 | 0.2009 | OSR28 | 9 | 85.4 | 8 | 0.6930 | 0.6402 |
| RM282 | 3 | 55.8 | 11 | 0.7563 | 0.7327 | RM5384 | 9 | 90.7 | 3 | 0.1558 | 0.1465 |
| RM338 | 3 | 61.9 | 4 | 0.5210 | 0.4064 | RM1013 | 9 | 93.5 | 4 | 0.6526 | 0.5890 |
| RM218 | 3 | 67.8 | 7 | 0.5194 | 0.4403 | RM7492 | 10 | 0.0 | 9 | 0.6989 | 0.6695 |
| RM232 | 3 | 76.7 | 7 | 0.6692 | 0.6102 | RM7545 | 10 | 7.6 | 19 | 0.8915 | 0.8841 |
| RM7403 | 3 | 82.3 | 6 | 0.0618 | 0.0612 | RM6646 | 10 | 13.3 | 6 | 0.6493 | 0.6051 |
| RM6266 | 3 | 94.9 | 4 | 0.5802 | 0.5034 | RM244 | 10 | 15.0 | 4 | 0.4505 | 0.3743 |
| RM7097 | 3 | 115.6 | 7 | 0.6704 | 0.6063 | RM216 | 10 | 24.8 | 4 | 0.5578 | 0.4592 |
| RM135 | 3 | 120.4 | 7 | 0.6162 | 0.5826 | RM311 | 10 | 25.2 | 4 | 0.6309 | 0.5559 |
| RM168 | 3 | 122.8 | 6 | 0.5506 | 0.4505 | RM184 | 10 | 41.6 | 5 | 0.5550 | 0.4814 |
| RM186 | 3 | 127.4 | 4 | 0.3644 | 0.3126 | RM1125 | 10 | 46.8 | 8 | 0.7982 | 0.7681 |
| RM16 | 3 | 131.5 | 4 | 0.0818 | 0.0806 | RM258 | 10 | 48.8 | 4 | 0.4674 | 0.3764 |
| RM5475 | 3 | 137.9 | 11 | 0.8042 | 0.7776 | RM5629 | 10 | 53.6 | 5 | 0.5361 | 0.4545 |
| RM416 | 3 | 140.1 | 4 | 0.5206 | 0.4056 | RM6100 | 10 | 53.9 | 4 | 0.1398 | 0.1362 |
| RM6712 | 3 | 158.2 | 9 | 0.7326 | 0.6908 | RM1108 | 10 | 55.3 | 2 | 0.0208 | 0.0206 |
| RM448 | 3 | 161.3 | 6 | 0.6196 | 0.5488 | RM3773 | 10 | 58.9 | 8 | 0.7495 | 0.7099 |
| RM148 | 3 | 191.6 | 5 | 0.5811 | 0.4939 | RM269 | 10 | 69.6 | 6 | 0.5618 | 0.4920 |
| RM307 | 4 | 0.0 | 5 | 0.5136 | 0.4201 | RM5352 | 10 | 71.4 | 4 | 0.2110 | 0.1963 |
| RM335 | 4 | 5.4 | 9 | 0.6404 | 0.5743 | RM304 | 10 | 73.0 | 2 | 0.0208 | 0.0206 |
| RM518 | 4 | 7.9 | 8 | 0.7645 | 0.7265 | RM171 | 10 | 73.0 | 3 | 0.4835 | 0.3764 |
| RM3471 | 4 | 16.7 | 7 | 0.7320 | 0.6846 | RM6160 | 10 | 81.0 | 3 | 0.1006 | 0.0972 |
| RM4835 | 4 | 18.3 | 4 | 0.1034 | 0.1011 | RM590 | 10 | 83.3 | 4 | 0.4913 | 0.3899 |
| RM5687 | 4 | 25.4 | 9 | 0.6103 | 0.5883 | RM333 | 10 | 110.4 | 2 | 0.0208 | 0.0206 |
| RM6314 | 4 | 41.5 | 6 | 0.5283 | 0.4366 | RM286 | 11 | 0.1 | 5 | 0.5281 | 0.4279 |
| RM471 | 4 | 53.8 | 7 | 0.1598 | 0.1572 | RM6327 | 11 | 1.7 | 8 | 0.7397 | 0.6943 |
| RM5951 | 4 | 56.1 | 4 | 0.5206 | 0.4056 | RM1240 | 11 | 6.5 | 11 | 0.8109 | 0.7876 |
| RM142 | 4 | 60.2 | 6 | 0.4370 | 0.3913 | RM7557 | 11 | 9.2 | 3 | 0.4386 | 0.3512 |
| RM6997 | 4 | 62.1 | 10 | 0.8051 | 0.7771 | RM1812 | 11 | 10.3 | 3 | 0.1006 | 0.0972 |
| RM7563 | 4 | 68.3 | 6 | 0.6689 | 0.6097 | RM6544 | 11 | 19.8 | 3 | 0.1558 | 0.1465 |
| RM6114 | 4 | 72.0 | 8 | 0.6600 | 0.6178 | RM3133 | 11 | 32.7 | 4 | 0.1389 | 0.1338 |
| RM6589 | 4 | 85.2 | 6 | 0.6535 | 0.5891 | RM167 | 11 | 37.5 | 5 | 0.5146 | 0.4632 |
| RM317 | 4 | 96.0 | 7 | 0.4361 | 0.4047 | RM3701 | 11 | 45.3 | 9 | 0.7439 | 0.7057 |
| RM6089 | 4 | 97.7 | 5 | 0.5751 | 0.4853 | RM7391 | 11 | 54.3 | 2 | 0.0208 | 0.0206 |
| RM3513 | 4 | 99.6 | 5 | 0.5066 | 0.4233 | RM7303 | 11 | 64.2 | 2 | 0.0308 | 0.0303 |
| RM3836 | 4 | 108.2 | 7 | 0.5591 | 0.5303 | RM7120 | 11 | 66.6 | 4 | 0.5241 | 0.4250 |
| RM280 | 4 | 128.9 | 4 | 0.2568 | 0.2333 | RM287 | 11 | 68.6 | 5 | 0.6583 | 0.6058 |
| RM559 | 4 | 129.6 | 7 | 0.6103 | 0.5798 | RM457 | 11 | 78.8 | 2 | 0.0220 | 0.0217 |
| RM349 | 4 | 146.8 | 7 | 0.6949 | 0.6423 | RM5349 | 11 | 79.1 | 4 | 0.5474 | 0.4858 |
| RM348 | 4 | 160.8 | 4 | 0.2090 | 0.1946 | RM209 | 11 | 84.7 | 3 | 0.1194 | 0.1144 |
| RM1182 | 5 | 3.0 | 5 | 0.3730 | 0.3297 | RM21 | 11 | 85.7 | 6 | 0.3508 | 0.3304 |
| RM153 | 5 | 3.0 | 6 | 0.6969 | 0.6433 | RM7170 | 11 | 101.9 | 8 | 0.7692 | 0.7363 |
| RM122 | 5 | 3.0 | 4 | 0.0818 | 0.0806 | RM206 | 11 | 102.9 | 10 | 0.8284 | 0.8069 |
| RM159 | 5 | 5.4 | 6 | 0.6909 | 0.6450 | RM7163 | 11 | 112.4 | 2 | 0.0208 | 0.0206 |
| RM267 | 5 | 25.0 | 6 | 0.5834 | 0.5069 | RM6293 | 11 | 117.3 | 4 | 0.4029 | 0.3440 |
| RM437 | 5 | 31.5 | 3 | 0.0414 | 0.0410 | RM224 | 11 | 120.1 | 9 | 0.6927 | 0.6695 |
| RM3193 | 5 | 36.4 | 6 | 0.6123 | 0.5377 | RM20 | 12 | 3.2 | 5 | 0.4053 | 0.3818 |
| RM574 | 5 | 41.0 | 4 | 0.4448 | 0.3632 | RM19 | 12 | 20.9 | 4 | 0.3311 | 0.2940 |
| RM249 | 5 | 50.2 | 6 | 0.5491 | 0.4830 | RM247 | 12 | 26.7 | 9 | 0.6970 | 0.6495 |
| RM6082 | 5 | 53.5 | 8 | 0.6644 | 0.5970 | RM6296 | 12 | 26.7 | 2 | 0.0208 | 0.0206 |
| RM598 | 5 | 62.7 | 5 | 0.5086 | 0.4085 | RM7619 | 12 | 38.1 | 2 | 0.0208 | 0.0206 |
| RM473B | 5 | 78.7 | 13 | 0.8692 | 0.8574 | RM512 | 12 | 39.4 | 2 | 0.0208 | 0.0206 |
| RM164 | 5 | 91.4 | 8 | 0.5723 | 0.5535 | RM5746 | 12 | 39.4 | 10 | 0.8603 | 0.8439 |
| RM188 | 5 | 95.3 | 9 | 0.6116 | 0.5431 | RM277 | 12 | 48.2 | 3 | 0.2232 | 0.2025 |
| RM161 | 5 | 96.9 | 9 | 0.7497 | 0.7132 | RM1337 | 12 | 51.5 | 6 | 0.5358 | 0.4403 |
| RM305 | 5 | 96.9 | 6 | 0.3135 | 0.2926 | RM511 | 12 | 59.8 | 5 | 0.7087 | 0.6591 |
| RM3170 | 5 | 115.4 | 6 | 0.4443 | 0.4260 | RM500 | 12 | 60.2 | 3 | 0.1558 | 0.1465 |
| RM480 | 5 | 130.6 | 4 | 0.6090 | 0.5320 | RM1246 | 12 | 65.3 | 4 | 0.3202 | 0.2902 |
| RM5818 | 5 | 144.9 | 5 | 0.5780 | 0.5313 | RM7102 | 12 | 71.8 | 5 | 0.6283 | 0.5591 |
| RM8109 | 6 | 1.7 | 7 | 0.6209 | 0.5745 | RM309 | 12 | 73.0 | 5 | 0.6502 | 0.5898 |
| RM508 | 6 | 2.3 | 6 | 0.5131 | 0.4439 | RM6869 | 12 | 75.8 | 4 | 0.5175 | 0.4288 |
| RM510 | 6 | 11.5 | 6 | 0.6442 | 0.5822 | RM463 | 12 | 75.5 | 4 | 0.5987 | 0.5138 |
| RM225 | 6 | 26.2 | 9 | 0.7722 | 0.7402 | RM3331 | 12 | 89.5 | 8 | 0.5722 | 0.5341 |
| RM405 | 6 | 28.6 | 5 | 0.4889 | 0.4367 | RM270 | 12 | 91.3 | 5 | 0.5121 | 0.4692 |
| RM2126 | 6 | 32.7 | 5 | 0.6080 | 0.5310 | RM5479 | 12 | 95.4 | 13 | 0.8270 | 0.8048 |
| RM50 | 6 | 32.7 | 5 | 0.4086 | 0.3802 | RM17 | 12 | 107.4 | 10 | 0.8222 | 0.7983 |
| RM276 | 6 | 33.5 | 8 | 0.7151 | 0.6680 | RM12 | 12 | 107.4 | 11 | 0.5324 | 0.4300 |
| RM314 | 6 | 33.6 | 6 | 0.4071 | 0.3856 |  |  |  |  |  |  |
| **Total Alleles** | |  | | | | | | | 1559 |  |  |
| **Mean** | |  |  |  |  |  |  |  | 5.93 | 0.5265 | 0.4776 |
