# Supplementary material for: Time-Course Association Mapping of the Grain-Filling Rate in Rice (Oryza sativa L.)
Source: PLoS One. 2015 Mar 19;10(3):e0119959. doi: 10.1371/journal.pone.0119959 (PMC4366047; doi:10.1371/journal.pone.0119959)
Supplement: S3 Table — Bold alleles represent that they were alleles with positive phenotypic effects on top three GFR at each stage across two years. (DOC) [file pone.0119959.s003.doc]

**S3 Table. Allelic variation of the loci showing positive allele effects on the top three GFR at five stages in the year of 2011 and 2012.**

| **Grain filling stage** | **Locus-allele** | **Phenotypic effect value** | | | **Typical carrier variety** |
| --- | --- | --- | --- | --- | --- |
|  |  | **2011** | **2012** | **Average** |  |
| 7DAF | **RM3170-160** | 0.588 | 0.563 | 0.576 | Nannongjing62401 |
| 7DAF | RM3170-170 | 0.302 | 0.47 | 0.386 | Hongmangshajing |
| 7DAF | RM3170-180 | 0.025 | 0.051 | 0.038 | Ningjing1 |
| 7DAF | RM3170-155 | 0.045 | 0.023 | 0.034 | Nantouzhong |
| 7DAF | RM3170-140 | -0.055 | -0.169 | -0.112 | Cuganhuangdao |
| 7DAF | RM3170-165 | -0.096 | -0.104 | -0.1 | Manyedao |
| 7DAF | **RM6266-145** | 0.516 | 0.612 | 0.564 | Nannongjing62401 |
| 7DAF | RM6266-140 | 0.289 | 0.425 | 0.357 | Wanhuangdao |
| 7DAF | RM6266-160 | 0.022 | 0.029 | 0.026 | Xudao4 |
| 7DAF | RM6266-155 | -0.165 | -0.179 | -0.172 | Sanbailitou |
| 7DAF | **RM480-195** | 0.527 | 0.453 | 0.49 | Wumangzaodao |
| 7DAF | RM480-200 | 0.209 | 0.275 | 0.242 | Xianhui429 |
| 7DAF | RM480-225 | -0.031 | -0.053 | -0.042 | Suzhouqing |
| 7DAF | RM480-215 | -0.089 | -0.049 | -0.069 | 5jing20 |
| 14DAF | **RM528-135** | 0.605 | 0.805 | 0.705 | Tongjing 109 |
| 14DAF | **RM528-245** | 0.509 | 0.631 | 0.57 | Wumangzaodao |
| 14DAF | RM528-220 | 0.351 | 0.126 | 0.239 | Nannongjing62401 |
| 14DAF | RM528-200 | -0.374 | -0.161 | -0.268 | Hongmangshajing |
| 14DAF | RM528-185 | -0.254 | -0.366 | -0.31 | Wanmandao |
| 14DAF | **RM309-160** | 0.262 | 0.71 | 0.486 | Baoxintaihuqing |
| 14DAF | RM309-180 | 0.149 | 0.324 | 0.237 | Nannongjing62401 |
| 14DAF | RM309-165 | 0.307 | 0.107 | 0.207 | Cuganhuangdao |
| 14DAF | RM309-175 | -0.014 | -0.03 | -0.022 | Xudao3 |
| 14DAF | RM309-170 | -0.188 | -0.105 | -0.147 | Hongmangshajing |
| 21DAF | **RM5818-150** | 0.596 | 0.562 | 0.579 | Shuijingbaidao |
| 21DAF | **RM5818-155** | 0.594 | 0.471 | 0.533 | Erlibie, Shengtangqing2 |
| 21DAF | RM5818-145 | 0.276 | 0.222 | 0.249 | Kongqueqing |
| 21DAF | RM5818-160 | -0.098 | -0.106 | -0.102 | Tongjing 109 |
| 21DAF | RM5818-140 | -0.389 | -0.305 | -0.347 | Baoxintaihuqing |
| 21DAF | **RM224-135** | 0.202 | 0.428 | 0.315 | Zaoshirihuangdao |
| 21DAF | RM224-120 | 0.118 | 0.23 | 0.174 | Xiaobaiyedao |
| 21DAF | RM224-125 | 0.089 | 0.109 | 0.099 | Aizhongluohanhuang |
| 21DAF | RM224-130 | 0.011 | 0.007 | 0.009 | Baimangnuo |
| 21DAF | RM224-140 | -0.017 | -0.027 | -0.022 | Wanluli |
| 21DAF | RM224-150 | -0.026 | -0.02 | -0.023 | Nantouzhong |
| 21DAF | RM224-160 | -0.081 | -0.057 | -0.069 | Hongmangshajing |
| 21DAF | RM224-145 | -0.113 | -0.105 | -0.109 | Erheidao |
| 21DAF | RM224-155 | -0.601 | -0.545 | -0.573 | Daniaodao |
| 28DAF | **RM72-205** | 0.609 | 0.406 | 0.508 | Laolaihong |
| 28DAF | RM72-180 | 0.029 | 0.013 | 0.021 | Yanglingdao |
| 28DAF | RM72-210 | 0.008 | 0.002 | 0.005 | Sidao10 |
| 28DAF | RM72-170 | -0.106 | -0.284 | -0.195 | Wanhuangdao |
| 28DAF | **RM263-175** | 0.67 | 0.319 | 0.495 | Laolaihong |
| 28DAF | RM263-160 | 0.115 | 0.129 | 0.122 | Yanglingdao |
| 28DAF | RM263-170 | -0.011 | -0.005 | -0.008 | Xiangzhunuo |
| 28DAF | RM263-190 | -0.412 | -0.244 | -0.328 | Baikenuo |
| 28DAF | **RM525-145** | 0.286 | 0.356 | 0.321 | Wumangzaodao |
| 28DAF | RM525-135 | 0.146 | 0.16 | 0.153 | Laolaihong |
| 28DAF | RM525-140 | -0.023 | -0.011 | -0.017 | Yanglingdao |
| 28DAF | RM525-110 | -0.215 | -0.461 | -0.338 | Baoxintaihuqing |
| 35DAF | **RM511-145** | 0.062 | 0.098 | 0.08 | Zhen9424 |
| 35DAF | **RM511-135** | 0.038 | 0.075 | 0.057 | Taijing 9 xuan |
| 35DAF | RM511-130 | 0.049 | 0.025 | 0.037 | Cuyingwanyangdao |
| 35DAF | RM511-140 | -0.034 | -0.012 | -0.023 | Cbao |
| 35DAF | RM511-125 | -0.097 | -0.027 | -0.062 | Wanmandao |
| 35DAF | **RM1013-160** | 0.104 | 0.036 | 0.07 | Kaiqing, Diantun502xuanzao |
| 35DAF | RM1013-155 | -0.003 | -0.009 | -0.006 | Longgouzhong |
| 35DAF | RM1013-150 | -0.018 | -0.006 | -0.012 | Huaidao9 |
| 35DAF | RM1013-145 | -0.177 | -0.221 | -0.199 | Shuijingbaidao |

Bold alleles represent that they were alleles with positive phenotypic effects on top three GFR at each stage across two years.
